# Umbrella effect of monitoring protocols for mammals in the Northeast US

**DOI:** 10.1038/s41598-022-05791-x

**Published:** 2022-02-03

**Authors:** Alessio Mortelliti, Allison M. Brehm, Bryn E. Evans

**Affiliations:** Department of Wildlife, Fisheries, and Conservation Biology, University of Maine, 5755 Nutting Hall, Orono, ME 04469 USA

**Keywords:** Ecology, Conservation biology

## Abstract

Developing cost-effective monitoring protocols is a priority for wildlife conservation agencies worldwide. In particular, developing protocols that cover a wide range of species is highly desirable. Here we applied the ‘umbrella species’ concept to the context of ecological monitoring; specifically testing the hypothesis that protocols developed for the American marten would contextually allow detecting occupancy trends for 13 other mammalian species (i.e., an umbrella effect). We conducted a large-scale four-year camera trapping survey across a gradient of forest disturbance in Maine, USA. We sampled 197 sites using a total of 591 cameras and collected over 800,000 photographs to generate detection histories for the most common terrestrial species. By combining multi-season occupancy modelling and power analyses, we estimated the required sampling effort to detect 10%, 25% and 50% declines in the fourteen species. By conducting a spatially explicit comparison of sampling effort, we found evidence that monitoring protocols for American marten would provide an umbrella effect for up to 11 other mammal species. The capacity of the umbrella effect varied among species, with fisher, snowshoe hare, red squirrel, and black bear consistently covered under several scenarios. Our results support the application of the umbrella species concept to monitoring (here defined as ‘umbrella monitoring species’), providing empirical evidence for its use by management agencies.

## Introduction

Monitoring, defined as the collection of repeated observations or measurements to evaluate changes in conditions and progress towards meeting a management objective^1^, is critical to the management of natural resources. It provides crucial information on the status of species, populations, and communities; allows assessment of the consequences of conservation actions; and constitutes an important step in adaptive management^2–4^. Indeed monitoring (and research) activities account for up to 50% of the budget allocated to conservation plans for threatened species^5^.

Developing and implementing monitoring programs is challenging for many reasons. First, unlike ‘surveillance’ programs (i.e., collections of repeated observations), statistically robust monitoring programs often require a combination of pilot field studies with power analysis and optimization algorithms^1,3,4^. Second, the definition of good and sound management objectives is critical to the success of a monitoring program. Third, the limited resources available for conservation work requires stakeholders to either (1) prioritize target species to monitor, or (2) seek strategies to optimize monitoring programs—such as identifying species whose monitoring would also enable detecting population trends for other species. The latter approach is analogous to the ‘umbrella species’ concept in conservation planning. An umbrella species is a species whose conservation confers protection to a large number of naturally co-occurring species^6^. By extension, and in the context of ecological monitoring, we propose an ‘umbrella monitoring species’ be a species whose monitoring protocols enable detecting trends for other species. As an example, a point-count based monitoring program designed for a rare forest songbird may also work as a monitoring program to detect trends in occupancy for many other birds in the community.

Clearly, the critiques to the umbrella species conservation concept apply to the monitoring equivalent^7–9^. Nevertheless, when developing a monitoring protocol, one should always test if and to what extent other species may be covered, since small tweaks to the program may allow conservation practitioners to derive broader benefits.

Developing monitoring protocols for mammals is challenging because of the low detectability and species-specificity in the monitoring methods. Nevertheless, camera traps are becoming the most cost-effective tool for large-scale and long-term population monitoring, with the intrinsic advantage that they allow the simultaneous detection of a wide-array of mammalian species^10–13^. Further, data collected by camera trapping can be analyzed in an occupancy modeling framework, which allows researchers to tackle the issue of false absences, and also to conduct power analysis and identify optimal sampling effort for a given management objective^14–16^. While several papers have focused on developing optimal monitoring protocols, including for camera traps^17–19^, an empirical assessment of the extent and the conditions under which one can benefit from an umbrella effect is lacking. Our goal here is to contribute to filling this knowledge gap. Specifically, our aims are to:

*Objective #1* To develop optimal monitoring protocols for fourteen mammalian species across the state of Maine (northeast USA), in relation to land-use.

*Objective #2* To assess if, to what extent, and under which conditions, a monitoring protocol for one species would allow concurrent monitoring of other species. We elected to focus on the American marten (*Martes americana*) as a potential ‘umbrella monitoring species’ due to its conservation and flagship status^20^, and due to its important role in forest ecosystems as both predator and prey^21^.

To achieve these objectives we established a field study across the northern two-thirds of the state of Maine, wherein we balanced survey effort for terrestrial mammals across a gradient of forest disturbance intensity and latitude. Mammalian distribution data was collected through transects of camera traps optimized for carnivore species^22–24^ across 197 survey sites, sampled over a 4-year period.

## Results

**Figure 1:**
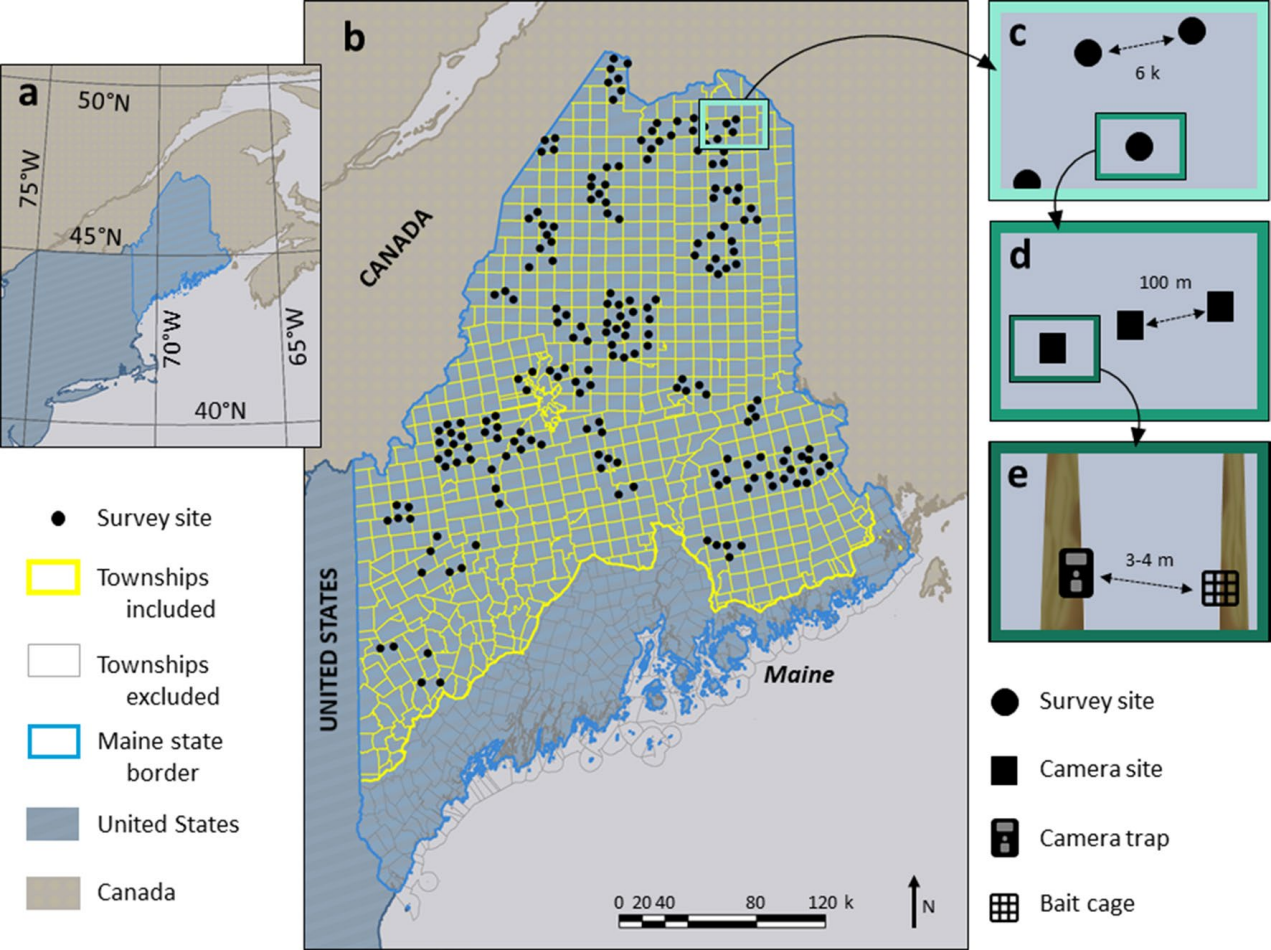
Map of the study area in Maine, in the northeastern United States (a). Survey sites were concentrated in the northern two-thirds of the state, enabling inference for monitoring protocols across 735 townships (**b**). Each survey site was placed a minimum of 6 k from any other site (**c**) and was composed of three camera traps arranged in a linear array (**d**). Each camera was set facing a tree with bait and lure (**e**). The map was created using ArcMap version 10.7.0.104507, Esri 2018, Advanced License.

In the period from June 2017 to October 2020 we deployed trail cameras at 197 sites (each comprised of three cameras in a linear transect) across the state of Maine (Fig. 1, Fig. S1). Each site was active for 2–4 weeks, and replicated in at least one summer season and the following winter season (up to all seven seasons for a subset of sites). Throughout the four-years of surveys, we collected over 800,000 photographs, resulting in the detection of a total of 27 mammalian species. The most detected species were the red squirrel (*Tamiasciurus hudsonicus*; with 5718 independent detections), snowshoe hare (*Lepus canadensis*; 3827), black bear (*Ursus americanus*; 1141), fisher (*Pekania pennanti*; 1112) and American marten (1086). Other species with sufficient detection histories for inclusion in our analyses were moose (*Alces alces*; 728), white-tailed deer (*Odocoileus virginianus*; 675), short-tailed weasels (*Mustela erminea*; 643), raccoon (*Procyon lotor*; 444), coyote (*Canis latrans*; 435), red fox (*Vulpes vulpes*; 145), lynx (*Lynx canadensis*; 120), porcupine (*Erethizon dorsatum*; 91) and bobcat (*Lynx rufus*; 58). Rarely detected species, without sufficient data for occupancy modeling, included Virginia opossum (*Didelphis virginiana*; with 1 detection), gray fox (*Urocyon cinereoargenteus*; 2), and river otter (*Lontra canadensis*; 13).

### Factors affecting the probability of detection

**Figure 2:**
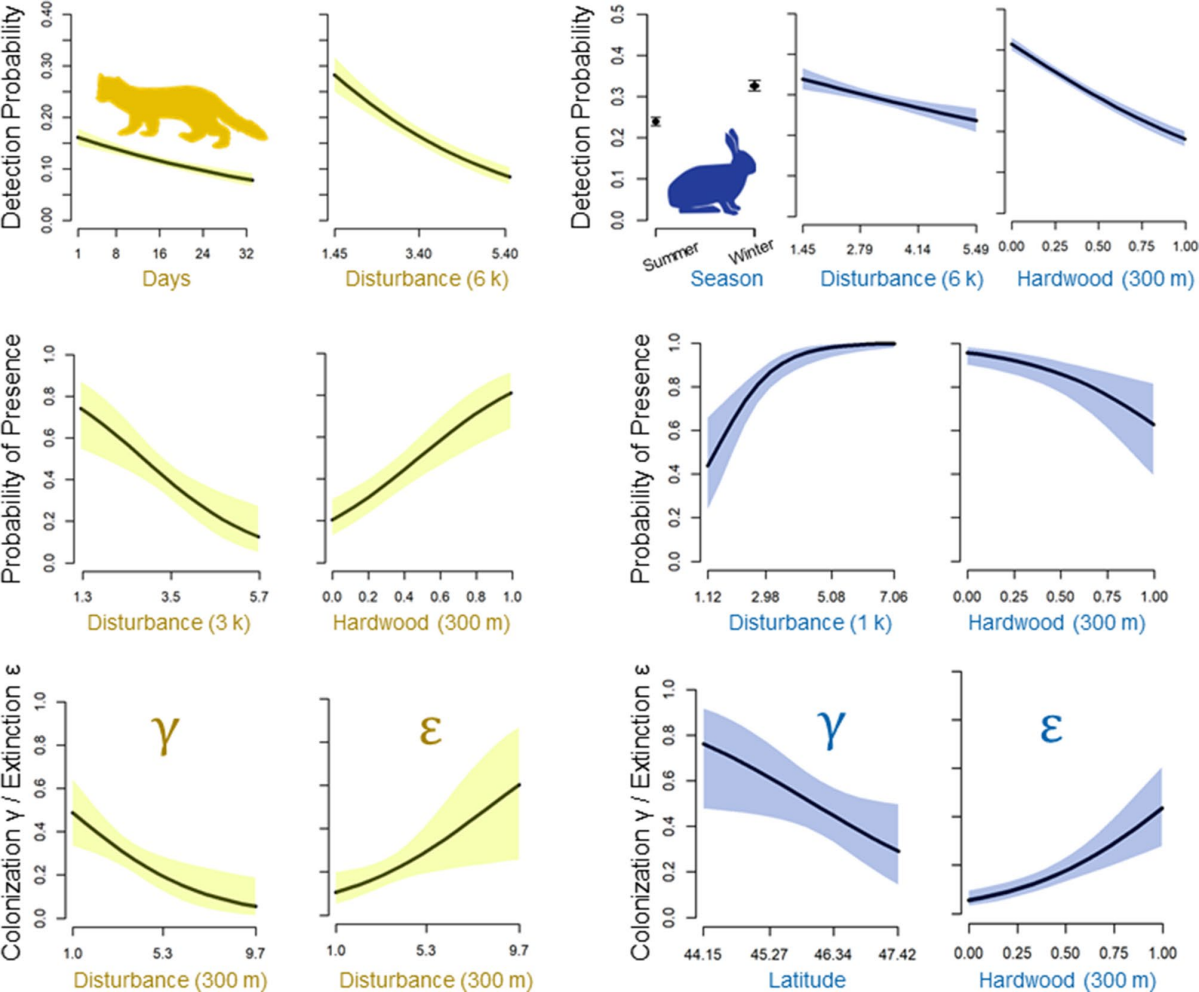
Predictions from the top ranked multi-season occupancy models from 197 camera survey stations deployed in Maine, USA. (Left) Marten (*Martes americana*) probability of detection is negatively related to forest disturbance intensity (6 k buffer), and days since the start of the survey. Marten initial occupancy (probability of presence) is negatively related to disturbance (3 k), and positively related to the proportion of hardwood (300 m buffer). Marten local extinction probability (ε) is more likely with increased disturbance (300 m buffer), and colonization probability is less likely in more disturbed areas. (Right) Snowshoe hare (*Lepus americanus*) probability of detection is higher in winter and decreases with disturbance intensity (6 k buffer) and the proportion of hardwood (300 m buffer). Snowshoe hare initial occupancy was negatively related to the proportion of hardwood and positively related to disturbance (1 k buffer). The probability of colonization (γ) decreases with latitude and the probability of local extinction increases with the proportion of hardwood (300 m buffer). The shaded are includes the 95% CI. Results for other species are shown in Fig. S2.

Detectability was higher in the winter season for fisher, lynx, snowshoe hare and porcupine, whereas it was higher in summer for black bear, raccoon, moose and red squirrel (Table 1, Fig. 2 and Fig. S2). In two cases (marten and short-tailed weasel) detectability decreased over the duration of the surveys (negative effect of the variable ‘days’). The effects of habitat variables (forest disturbance and proportion of hardwood) matched the habitat requirements for each species. For example, detectability was higher in less disturbed forest for marten and fisher, whereas it was higher in recently disturbed stands for lynx and moose (Fig. S2). Latitude affected detection in eight species, with the probability of detection decreasing with latitude in the cases of coyote, red fox, raccoon, bobcat, white tailed deer and porcupine, and increasing in the case of black bear and the lynx (Table 1, Fig. 2 and Fig. S2).

**Table 1:** Ranking of multi-season occupancy models within 2 ΔAIC of the top model for all 14 species.

Species	Model description	ΔAIC	*R*^*2*^
**American marten**	Ψ(Prop. hard. 300 m + Dist. 3 k)γ(Dist. 300 m)ε(Dist. 300 m)p(days + Dist. 6 k)	0.000	0.625
**Fisher**			
	Ψ(.)γ(Prop. hard. 300 m)ε(Dist. 6 k)p(Dist. 6 k + season + Prop. hard. 1 k)	0.000	0.685
**Short tailed weasel**			
	Ψ(1)γ(Prop. hard. 1 k + Dist. 1 k)ε(.)p(days^2^)	0.000	0.463
	Ψ(1)γ(Prop. hard. 1 k + Dist. 1 k)ε(Prop. hard. 300 m)p(days^2^)	0.489	0.468
	Ψ(1)γ(Prop. hard. 1 k + Dist. 1 k)ε(Dist. 6 k)p(days^2^)	1.261	0.465
	Ψ(1)γ(Prop. hard. 1 k + Dist. 1 k)ε(Dist. 3 k)p(days^2^)	1.550	0.465
	Ψ(1)γ(Prop. hard. 1 k + Dist. 1 k)ε(Dist. 300 m)p(days^2^)	1.680	0.464
**Black bear**			
	Ψ(.)γ(.)ε(Prop. hard. 1 k)p(LAT + season + Prop. hard. 1 k)	0.000	0.570
**Lynx**			
	Ψ(Prop. hard. 1 k + Dist. 3 k)γ( Prop. hard. 300 m)ε(LAT)p(LAT + Dist. 300 m + season)	0.000	0.287
	Ψ(Prop. hard. 1 k + Dist. 3 k)γ( Prop. hard. 300 m)ε(Dist. 300 m + LAT)p(LAT + Dist. 300 m + season)	0.594	0.292
**Bobcat**			
	Ψ(LAT + Dist. 6 k)γ(.)ε(Prop. hard. 300 m)p(Dist. 300 m + LAT)	0.000	0.309
	Ψ(LAT + Dist. 6 k)γ(.)ε(.)p(Prop.hard. 1 k)	1.281	0.304
	Ψ(LAT + Dist. 6 k)γ(.)ε(LAT.)p(Dist. 300 m + LAT)	1.469	0.303
	Ψ(LAT + Dist. 6 k)γ(.)ε(Dist. 1 k)p(Dist. 300 m + LAT)	1.810	0.302
	Ψ(LAT + Dist. 6 k)γ(.)ε(Dist. 300 m)p(Dist. 300 m + LAT)	1.819	0.302
	Ψ(LAT + Dist. 6 k)γ(.)ε(Dist. 3 k)p(Dist. 300 m + LAT)	1.987	0.301
**Coyote**			
	Ψ(Dist. 3 k)γ(LAT)ε(LAT)p(LAT)	0.000	0.226
**Red fox**			
	Ψ(Prop. hard. 1 k)γ(Dist. 3 k)ε(.)p(LAT)	0.000	0.372
	Ψ(Prop. hard. 1 k)γ(Dist. 3 k)ε(LAT)p(LAT)	0.948	0.375
	Ψ(Prop. hard. 1 k)γ(Dist. 3 k)ε(.hard300)p(LAT)	1.483	0.374
	Ψ(Prop. hard. 1 k)γ(Dist. 3 k)ε(.)p(Prop.hard. 1 k)	1.894	0.372
**Raccoon**			
	Ψ(Prop. hard. 1 k + LAT)γ(Prop. hard. 300 m + Dist. 1 k)ε(LAT)p(season + LAT)	0.000	0.346
**Deer**			
	Ψ(.)γ(Dist. 6 k)ε(Dist300 + LAT)p(LAT + Dist. 6 k + Prop. hard. 300 m)	0.000	0.490
**Moose**			
	Ψ(LAT)γ(.)ε(Dist. 1 k)p(Dist. 3 k + season)	0.000	0.631
	Ψ(LAT)γ(.)ε(Dist. 300 m + LAT)p(Dist. 3 k + season)	0.212	0.634
**Snowshoe hare**			
	Ψ(Prop. hard. 300 m + Dist. 1 k)γ(LAT)ε(Prop. hard. 300 m)p(Prop. hard. 300 m + Dist. 6 k + season)	0.000	0.922
**Red squirrel**			
	Ψ(1)γ(Prop. hard. 300 m + Dist. 3 k)ε(Prop. hard. 300 m)p(Prop. hard. 300 m + factor(season) + Dist. 3 k)	0.000	0.797
**Porcupine**			
	Ψ(Prop. hard. 1 k)γ(1)ε(.)p(season + LAT + Prop. hard. 1 k)	0.000	0.238
	Ψ(Prop. hard. 1 k)γ(1)ε(Dist. 6 k.)p(season + LAT + Prop. hard. 1 k)	0.015	0.246
	Ψ(Prop. hard. 1 k)γ(1)ε(LAT.)p(season + LAT + Prop. hard. 1 k)	1.223	0.241
	Ψ(Prop. hard. 1 k)γ(1)ε(Dist. 3 k.)p(season + LAT + Prop. hard. 1 k)	1.490	0.240
	Ψ(Prop. hard. 1 k)γ(1)ε(Prop. hard. 1 k)p(season + LAT + Prop. hard. 1 k)	1.513	0.240
	Ψ(Prop. hard. 1 k)γ(1)ε(Dist. 300 m.)p(season + LAT + Prop. hard. 1 k)	1.980	0.238
	Ψ(Prop. hard. 1 k)γ(1)ε(Dist. 1 k.)p(season + LAT + Prop. hard. 1 k)	1.990	0.238

Detection data were collected over four years at 197 camera trap stations in Maine, USA. Prop. hard. 300 m = proportion of hardwood within a 300 m radius buffer of the site; Prop. hard. 1 k = proportion of hardwood within 1 k of the site; Dist. 300 m = Intensity of forest disturbance within 300 m of the site; Dist. 1 k = Intensity of forest disturbance within 1 k of the site; Dist. 3 k = Intensity of forest disturbance within 3 k; Dist. 6 k = Intensity of forest disturbance within 6 k of the site; LAT = latitude; days = days since start of survey at a site (days^2^ = quadratic effect); season = winter or summer; ΔAIC = Delta Akaike Information Criterion; *R*^2^ = Nagelkerke’s R squared.

### Factors affecting occupancy and turnover (local colonization and extinction)

Factors affecting the probability of a site being occupied during the first season (ψ) and local colonization (γ) and extinction (ε) between seasons reflected the main habitat requirements for each species. As an example, both marten and white tailed deer were negatively associated with more highly disturbed forest areas (marten: negative relationship with ψ and γ and positive with ε; white tailed deer: negative relationship of disturbance with γ and positive relationship with ε). Alternatively, snowshoe hare, bobcat, lynx, moose and coyote, which can all benefit from early successional forest habitat, were positively associated with disturbed areas (Table 1, Fig. 2 and Fig. S2). The association with an increasing proportion of hardwood was positive (marten, fisher, porcupine, raccoon and red fox) or negative (lynx, short-tailed weasel, black bear red squirrel and snowshoe hare).

Occupancy and turnover varied with latitude for seven species, with both negative (coyote, bobcat, white-tailed deer, snowshoe hare and raccoon) and positive relationships (moose, lynx,) (Table 1, Fig. 2 and Fig. S2).

### Sampling effort

Required sampling efforts to detect a 10%, 25% or 50% decrease in occupancy varied considerably between species: red squirrel, bear, marten and fisher were the easiest to monitor and bobcat, lynx and porcupine required the most extensive sampling efforts (Fig. S3). For several species, such as the American marten, sampling effort clearly matched habitat requirements, with the lowest effort required in the most suitable habitat.

### Umbrella effect

**Figure 3:**
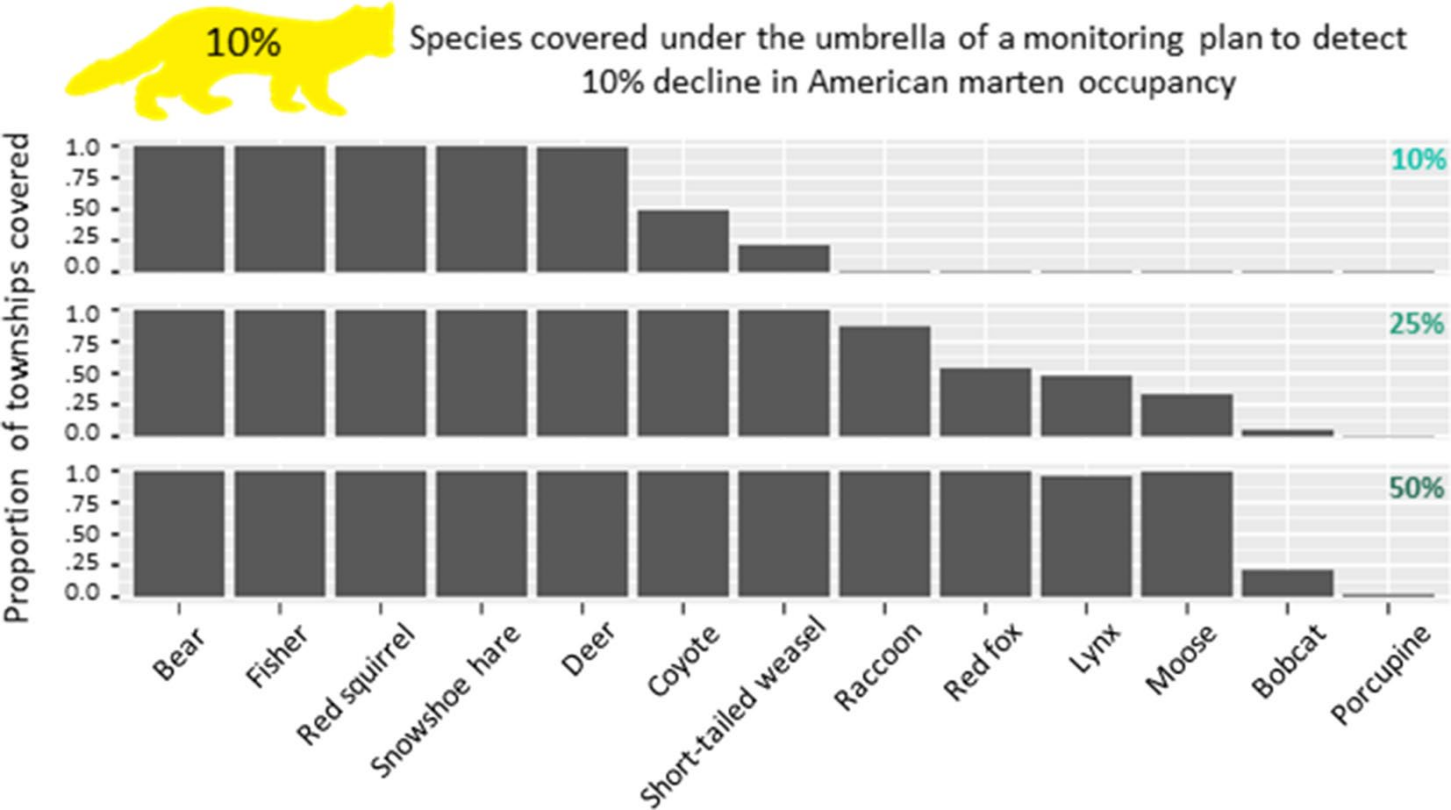
Umbrella effect (10% marten decline). The figure shows the proportion of townships per species that are covered under the 10% marten decline sampling protocol. The top figure shows a 10% decline for each species i.e. a 10% marten decline sampling effort would cover 100% of townships for the bear under a 10% change in bear occupancy scenario. The middle figure shows a 25% decline for each species i.e. a 10% marten decline sampling effort would cover approximately 50% of townships for the red fox under a 25% change in red fox occupancy scenario. The bottom figure shows a 50% decline for each species i.e. a 10% marten decline sampling effort would cover approximately 25% of townships for the bobcat under a 50% change in bobcat occupancy scenario.

**Figure 4:**
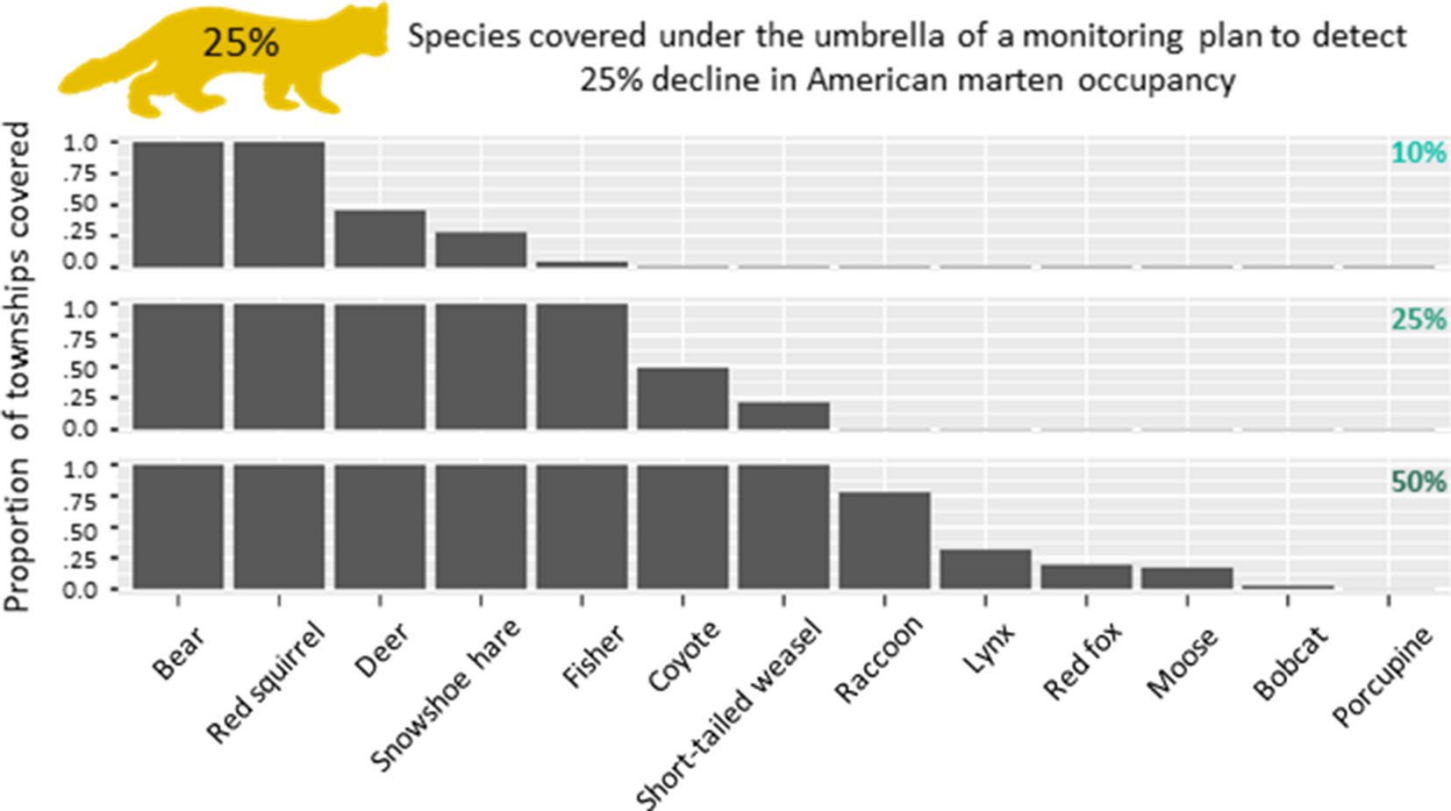
Umbrella effect (25% marten decline). The figure shows the proportion of townships per species that are covered under the 25% marten decline sampling protocol: top are covered at a 10% decline, middle at a 25% decline, and bottom at a 50% decline.

**Figure 5:**
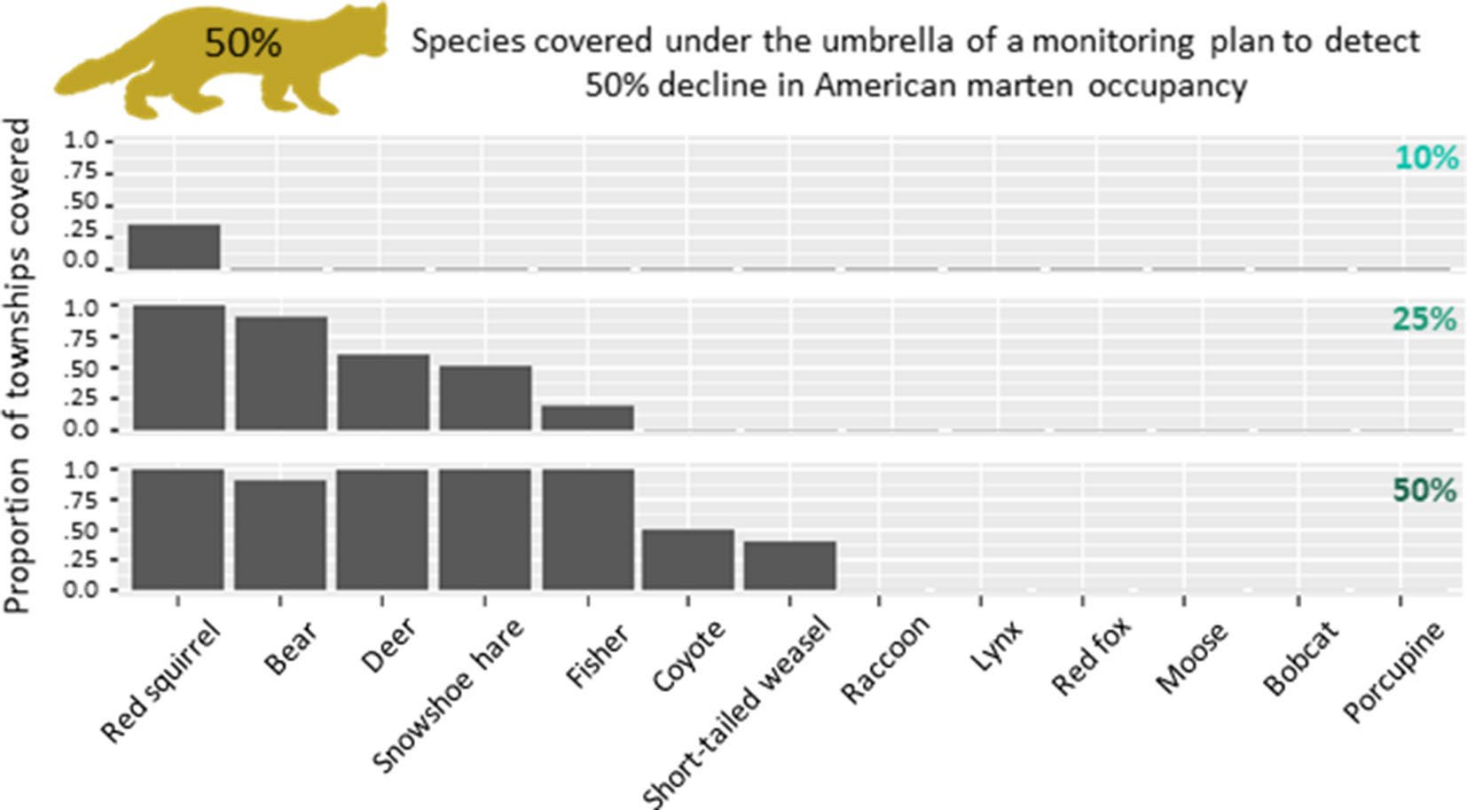
Umbrella effect (50% marten decline). The figure shows the proportion of townships per species that are covered under the 50% marten decline sampling protocol: top are covered at a 10% decline, middle at a 25% decline, and bottom at a 50% decline.

Our analyses show that up to 11 species benefit from an umbrella effect under a monitoring protocol designed for American marten (Figs. 3, 4, 5) at three different management objectives: the detection of 10%, 25%, and 50% declines in occupancy. The effect is consistent across management objectives in the case of several species (black bear, red squirrel, deer, fisher, and snowshoe hare), and encompasses up to 100% of the townships surveyed (Figs. 3, 4, 5). Some species (coyote, raccoon, and short-tailed weasel) show an umbrella effect when marten are monitored for high effort objectives (10% marten decline, Fig. 3) or when only a large detectable change in the non-target species is needed (i.e., 50% decline in red fox, lynx, and moose, Fig. 3). The least covered species were bobcat and porcupine, with the latter never being covered and the former being partly covered (approximately 25% of townships that could be included to detect a 10% marten decline would meet the requirements for detecting a 50% bobcat decline).

While the 10% management objective scenario shows the strongest umbrella effect (11 species fully covered to detect 50% decline, Fig. 3), these protocols require an extensive effort. On the opposite side, efforts required for a 50% marten management objective are low, but so is the umbrella effect (1 species covered at 10% decline, and 7 species covered at 50% decline detection, Fig. 5). A 25% management objective seems a good compromise in terms of feasibility (we consider 110–180 camera transects a reasonable survey effort, see Appendix S1) and effectiveness of the umbrella effect (range of species covered is 3–4 for 10% decline detection and 10–11 for 50% decline detection, Fig. 4).

## Discussion

Through a large-scale field study, we found evidence that developing camera trapping monitoring protocols for the American marten would provide an umbrella effect for up to 11 other species of mammals. The effectiveness of the umbrella effect varied among species, with fisher, snowshoe hare, red squirrel, and black bear consistently covered under several combinations of management objective and decline detection, whereas porcupine and bobcat were the least covered species. Besides varying among species, the effectiveness of the umbrella effect also varied among the several monitoring objectives considered. For example, when following the marten 25% decline management objective, which requires an intermediate sampling effort, we can assess the umbrella effect for also monitoring coyote at three levels of precision: (1) It would not be possible to detect a 10% decline of coyote, which requires more sampling effort than available in any townships. (2) There would be intermediate coverage for detecting a 25% coyote decline, with about half of the townships eligible for a marten monitoring protocol also covering coyote (see case study, Appendix S1). (3) There would be full coverage for detecting a 50% decline in the coyote occupancy patterns, where any townships included for a marten protocol will also cover coyote (Fig. 4). Our results thus show that by flexibly adjusting monitoring objectives, a wide range of species may be monitored through the same protocol.

Previous work on developing monitoring protocols through occupancy modelling focused both on single species^19,25^ or multiple species^26,27^, underscoring the importance of conducting rigorous power analyses. We advanced our knowledge on the topic of optimal monitoring by empirically testing for an umbrella effect, contextually showing the extent to which the approach is applicable to multi-species monitoring of the mammalian community. While the traditional application of the umbrella species focuses on area requirements^6^, we adapted this concept to the development of monitoring protocols (here defined as ‘umbrella monitoring species’). We emphasize that the species with the highest sampling requirement (i.e. total number of camera trapping transects for a given management objective) and the widest distribution overlap with other species would automatically act as an umbrella—however our results clearly show that developing monitoring protocols for the most difficult species may not be logistically feasible (see Fig. S3g as an example). By choosing a species such as the American marten, which requires intermediate sampling effort, we were able to show how some species are consistently covered (Figs. 3, 4, 5). Furthermore, we were able to show that with flexibility in determining monitoring objectives (such as adapting the targeted precision of detectable changes), the majority of other species may be also be covered. As an example, a protocol targeting a 25% decline in marten occupancy would allow the detection of a 50% decline across at least some portions of lynx occupancy (a species of conservation concern in the US^28^). Increasing the precision to detecting a 10% marten decline, while requiring a more intensive (yet potentially still feasible) protocol, would allow much better monitoring for lynx: 50% decline anywhere surveyed, and extensive areas detecting a 25% lynx occupancy decline. This tangible improvement may provide the necessary evidence to an agency tasked with deciding to make the additional investment (i.e., cover the additional cost of a 10% marten protocol over a 25% marten protocol) to maximize the multi-species coverage. Notably, these protocols would allow monitoring the lynx as well as its main prey (snowshoe hare) in their prime habitat, while simultaneously covering non-optimal habitat for the marten, which may be the first habitat to experience significant declines of the species.

Our occupancy results are in line with the known biology of the species and, in most cases, we found that required sampling effort matches the suitability of habitats, reflecting a positive association with average occupancy and detectability. As an example in the case of the marten, less disturbed areas^29^ and areas with a higher proportion of hardwood^30^ were characterized by higher detection and overall occupancy (higher initial occupancy, lower extinction and higher colonization), which was reflected by lower sampling effort required for these areas. Likewise, in the case of snowshoe hare, higher occupancy occurred in more disturbed areas and areas with lower proportion of hardwood (thus a higher proportion of conifers)^31^, which was reflected in the sampling protocols (Fig. S3k).

Our sampling encompassed a strong latitudinal gradient, which is clearly reflected in the occupancy patterns of several species. While coyote were captured throughout the state, this expanding species^32^ is more prevalent in the south. Likewise, average occupancy of deer and raccoon are higher in the southern part of the state, and bobcat are entirely absent from the northern part of the state. The opposite is true for moose (higher initial occupancy in the northern part of the state), and for lynx, which have not expanded into southern Maine.

To what extent may our findings be generalized? While it is likely that, with some modifications, the protocols we developed will be applicable to other regions in the Northeast US, it is possible that the umbrella monitoring effect itself will apply to other areas. Nevertheless, we emphasize that the magnitude and extent of coverage are context dependent, therefore additional data will need to be collected to calibrate the applicability of our findings elsewhere. Nevertheless, we emphasize that mammalian community structures are similar in other temperate systems in North America and Europe. Thus, we foresee extensive potential in seeking similar patterns elsewhere.

Our previous work has shown that the use of bait and lure clearly maximizes detection of carnivores (mustelids in particular) without affecting detection of non-target species^23^. A more neutral approach (no bait or lure) would likely work too, however in our case detection probabilities would have been extremely low, leading to very high sampling efforts^23^. Further, we emphasize that our sampling unit was a transect with three cameras, which maximizes detections and reduces the variance in parameter estimates^1,22^. While the approach may still work with a single camera, the benefits of the umbrella monitoring effect may be substantially reduced, thus we emphasize the importance of using multiple cameras deployed through a transect^22^.

We acknowledge that we did not examine all possible scenarios. As an example we limited our analyses to using the marten as an umbrella monitoring species, we did so based on its flagship status^20^ and on the fact that protocols were developed with this species in mind. Likewise, we kept the value of alpha constant to 0.05 and the desired power level to 0.8, which are widely used for power analyses^1,16,33^. Modifying these values would clearly impact the strength of the umbrella monitoring species effect.. As an example, a manager may decide to develop a 25% marten decline protocol, but contextually accept a higher risk of type I error for the lynx (e.g. risk of incorrectly detecting a decline), which would lead to an increase in the coverage of the umbrella effect for this species. For the sake of simplicity, we did not explore these scenarios, but encourage practitioners to do so depending on their specific management objectives.

Finally, we emphasize that we grouped townships that required similar sampling effort (Fig. S3, Appendix S1), which may lead to over-sampling in some areas (for example if townships requiring 15 transects are grouped with those requiring 20 transects); nevertheless we suggest that it is better to be conservative and over-sample, rather than the opposite. We also underscore the need to follow basic sampling design rules, such as making sure that the areas sampled are representative and biologically meaningful. When the predicted sampling effort is very low (i.e. black bear in Fig. S3h) this is a clear indication that a) one should aim for smaller detectable changes, such as 10% and b) one should still ensure reasonable effort commensurate with the size of the area monitored.

The sampling effort required to detect a specific trend in occupancy refers to a specific value of average ψ (calculated over a 4-year period). As an example, for the American marten a 25% decline protocol with a ψ of 0.6 and a *p* of 0.3, requires a sampling effort of eight transects (with an alpha of 0.05 and a power of 0.8). Should a practitioner develop a protocol for each value of probability of presence (ψ)? That is an option, however we argue that it is also possible to group sites into high, medium and low effort areas (which in this specific case correspond to low, medium and high average ψ areas) and develop protocols for these merged categories. Such an approach will allow practitioners to prioritize areas, so one can decide whether to invest in areas of critical habitat (here low effort) or whether to invest in monitoring areas that are less suitable, but which may provide the first signals of decreasing occupancy (high effort areas). In Appendix S1 we show an example of the implications of this type of choice for the umbrella effect of a 25% management objective protocol for marten to also detect a 25% decline for coyote.

Through our large-scale, four-year study focused on developing monitoring protocols for mammals in the Northeast, we found empirical evidence for an umbrella effect. Specifically, we found that a protocol focused on the American marten was able to cover up to 11 other species of mammals, demonstrating that multi-species monitoring is feasible and should be included in the portfolio of monitoring activities conducted by management agencies. While application of our results to other systems may require some calibration or completely new data collection, our results provide empirical evidence that such an approach is worth pursuing. Further, we encourage future efforts targeted towards identifying characteristics that make a species suitable to be used as a monitoring umbrella.

## Methods

### Study area

We conducted our research in Maine, a state in the northeastern United States, 45.2538° N, − 69.4455° W (Fig. 1a). Forests cover 17.6 million acres (89% of the state), with the majority of forests (94.9%) managed intensively for silviculture^34^. Forest management practices, typically partial-harvests, and natural disturbances have resulted in mostly mixed-age stands^35–37^. The north of the state falls in the Boreal Forest biogeographic region, dominated by spruces species (*Picea* sp.), balsam fir (*Abies balsamea*) and white pine (*Pinus strobus*). Dominant vegetation transitions to the Eastern deciduous species in the south, including maples (*Acer* sp.), birches (*Betula* sp.), and American beech (*Fagus grandifolia*)^38,39^. We distributed sampling effort across the upper 85% of the state, excluding the farthest south and east (coastal, less forested, and more developed) townships (Fig. 1b). The regional climate ranges from lows of − 10 °C to highs of 18.5 °C, with precipitation totaling 106 cm annually^40^.

### Study design

We designed a large-scale field study to understand occupancy patterns of the mammalian community across the range of forest disturbance regimes characteristic of Maine. We stratified our sampling to ensure balanced effort across all levels of forest disturbance as well as latitude within the study area, and tested for independence from the composition (i.e. softwood vs hardwood) of the forest stands (Fig. S1).

We calculated the forest disturbance intensity from Landsat^41^ image data that were processed using a novel method to combine multiple individual change-detection algorithms^42^. Image layers from 1948 to 2017 provided continuous raster indices describing the magnitude, duration and year of the most recent disturbance event at each 30 m pixel, calibrated specifically to New England forests. We used raster math to combine the time elapsed since, and magnitude of, the most recent disturbance into a score for each pixel across the state of Maine. From this layer we calculated the mean *disturbance index* at multiple spatial scales. These included: townships (for broad-scale study design), and buffers around each survey site (300 m, 1 k, 3 k and 6 k radius circular buffers).

We conducted camera trapping surveys at 197 locations distributed across the gradient of townships described above, each spaced a minimum distance of 6 k between stations to maximize independence between our detections. At each survey site we placed three Bushnell Trophy Cam E2/E3 passive infrared cameras (Overland Park, KS, USA) in a linear transect spaced 100 m apart. Through previous work^22^ we found that this configuration would maximize the probability of detection for most species^43,44^. See Supplementary Information Appendix S2 for example images of the 14 mammal species we included.

Each camera was set approximately 40 cm above the ground (or packed snow), facing towards a tree with a suet cage of meat bait attached at the base and scent lure applied at 2 m height^45,46^ (Fig. 1c–e). The relatively low height of the camera allowed us to maximize the detection of small animals such as weasels and squirrels while at the same time allowing the detection of larger animals such as moose and deer (a moose standing at 2 m from the camera is fully identifiable). The lure is designed to attract furbearers (skunk essence and Vaseline based, Kenduskeag, Maine, USA) and was applied to a tree in front of the camera at head height, and again at bait level. The bait consisted of American beaver (*Castor canadensis*) carcasses, which were cut to standard size ($\overline{x}$ = 0.22 kg) and protected by a suet cage approximately 2 m in front of the camera at an average height of 30 cm above the ground. In a previous study we demonstrated that the simultaneous use of bait and lure increased the detection probability of carnivore species without impacting non-carnivore mammals^23^. Specifically, we found that for mustelid species and for the black bear, the combined use of a bait and lure drastically increase detection, as an example the probability of detecting a marten increased from 0.1 to 0.5.We deployed survey stations in the summer season for > 15 days, and during the following winter season for > 15 days. Each year after the first we deployed a subset of sites for a third, fourth, up to seventh season. Seasons ran from June to September (summer) and January to April (winter), for the years 2017–2018, 2018–2019, 2019–2020, and summer 2020. During each season (i.e., the 2–4 weeks when the sites were active) the sites were not visited to minimize disturbance, and because there was no need to change the bait and lure as their functionality was preserved for several weeks^23^. All protocols followed the guidelines of the American Society for Mammalogy^47^ for free-ranging mammal research and were approved by the University of Maine IACUC Committee under Protocol #A2018-05-06.

### Occupancy models

We analyzed detection history data by fitting multi-season, single-species occupancy models using the unmarked package in R^48–50^. Occupancy modeling accounts for the potential bias introduced by false absences^51,52^ and allows inclusion of habitat variables that may influence the detection probability (*p*), as well as the probability of a site being occupied in the first season (*ψ*) and the subsequent local colonization (γ) and extinction probabilities (ε).

Detection histories from the three cameras in each transect were combined, with a confirmed image at 1 or more of the cameras in a 24-h period coded as 1 = detected, and no images recorded coded as 0 = no detection. Image data were processed using the Reconyx MapView Professional software (Holmen, WI, Version 3.7.2.2, Copyright RECONYX, Inc. 2005–2016) to link each image tag with the date and time that it was recorded. Every image was uploaded to a folder for that camera site, and tagged from a list of keywords identifying the cause of the trigger (e.g. “Setup”, “Canid_coyote”, “Bird_unknown”). The exported files were reviewed for accuracy and compiled prior to being pooled by 1) the three cameras at a site and 2) the date images were recorded.

The data were analyzed against the following variables: latitude, year and season of surveys, forest disturbance within 300 m, 1 k, 3 k and 6 k radius buffers around each site, and the proportion of hardwood trees within the immediate stand (data collected in the field), as well as 300 m and 1 k radius buffers (remotely sensed data^53^). We tested for correlation between our predictor variables and found that the correlation coefficient was less than 0.1 between covariates measuring different features (i.e., disturbance vs proportion of hardwood). As expected, correlation between different buffers of the same feature were high, for this reason these covariates were never used in the same model to avoid collinearity issues^54^.

We conducted the occupancy analyses following a forward stepwise approach and implemented four phases: We started by modeling detection probability as function of the following single predictors: latitude, year and season of surveys, forest disturbance (all buffers) and the proportion of hardwood trees (all buffers). We ranked models via Akaike Information Criterion^55^. If one or more model ranked within 2 ΔAIC of the top model, and was not modeling the same feature at a different spatial scale (e.g., disturbance at 1 k followed by disturbance at 300 m) additive models were also tested. We then retained the top model for the detection process as we repeated this step for the initial occupancy probability, then colonization, and finally extinction. For the latter three parameters the following predictors were used: latitude, forest disturbance (all buffers) and the proportion of hardwood trees (all buffers). We quantified model fit using the Nagelkerke’s Rsquared through R package unmarked.

### Sampling effort

We used the algorithms developed by Guillera-Arroita and Lahoz-Monfort^15^ to estimate the required sample size (e.g., the number and duration of camera deployments) for a given management objective. Specifically, Guillera-Arroita and Lahoz-Monfort^15^ showed that the number of sites to be surveyed to achieve a given power can be derived as a function of the significance level (alpha) and effect size (i.e. percent decline to be detected), given ψ (occupancy probability), *p* (detection probability), and the number of visits (survey days).

Management objectives (i.e., effect size or percent decline to be detected) were determined in collaboration with stakeholders (Maine Department of Inland Fisheries and Wildlife) and corresponded to detecting three degrees of decline in the occurrence of a species: 10% (minor), 25% (moderate) and 50% (extreme) throughout the northern two-thirds of the state (Fig. 1b). To reduce the possible combinations of monitoring protocols, we set the number of visits = 21, thus focusing our analyses on how the number of sites sampled impacts monitoring outcomes. We chose a 21 day duration since we found this would allow a very high (> 0.8–0.9^18,56^) cumulative probability (p*) of detection of the target species American marten, with p* = 1 − (1 − *p*)^k^ = the probability of detecting the species at least once. Alpha (the probability of a type I error, detecting a decline when a decline is not there) was set at 0.05 in all analyses^1^. We used the predicted occupancy probability averaged over four years as the initial occupancy value^15^ for sampling effort calculations. The predicted average occupancy was calculated^49,57^ as the average between ψ^1^, ψ^2^, ψ^3^ and ψ^4^, where ψ^1^ = occupancy in the first year, ψ^2^ = ψ^1^ * ε + (1 − ε) * γ; ψ^3^ = ψ^2^ * ε + (1 − ε) * γ; ψ^4^ = ψ^3^ * ε + (1 − ε) * γ.

We calculated sampling effort at the township level, which is a commonly used unit in Maine and can be scaled up to larger areas as needed. To match the estimates for survey effort to the correct spatial scale, we divided each township within our study area into grids, with cells of equal area to each of our buffer sizes (for example, a 3 k circular buffer around a survey station covers an area of approximately 28.27 k^2^, which translates to a square with sides of 5.317 k). We then obtained the average value of the disturbance index for all pixels in these grid cells and applied that as the value for the entire township. We repeated these calculations for the percentage of forest cover attributed to hardwood species from GAP/LANDFIRE^53^ at the equivalent of 300 m and 1 k radii buffer cells.

### Umbrella monitoring species

We next assessed the umbrella effect that monitoring American marten (hereafter the target), at each level of precision, would have for our array of non-target species. Non-target species included American black bear, bobcat, coyote, white-tailed deer, fisher, Canada lynx, moose, porcupine, raccoon, red fox, American red squirrel, short-tailed weasel, and snowshoe hare. As described above, we first estimated the sampling effort required for the detection of a minor (10%), moderate (25%) or extreme (50%) decline in the occurrence of the target throughout the northern two-thirds of the state.

After sampling effort was estimated at these three levels of change detection for the target, we identified, for each township in the study area, whether each non-target could be monitored using the same number of transects required for the target species in that township. If the sampling effort required for the non-target was less than or equal to the maximum number of transects required for the target, that township was scored as “in”, and if not, that township was scored as “out”. At each sampling effort for the target (10%, 25%, or 50% change detection), we implemented this process for each non-target species, setting the degree of decline detected to 10%, 25% or 50% change in occupancy. This allowed us to determine the following for each non-target species: if a monitoring protocol were established with enough effort to detect a 25% (or 10% or 50%) change in occupancy of the target species, in what percent of the sampling area would we be able to detect a 10% change, 25% change, or 50% change in occupancy for this non-target?

For these analyses we focused on areas where American marten occupancy over a four-year period was estimated to be 0.2 or higher, to avoid focusing on poor marten habitat. This removed only five townships from the analyses. Among the remaining 730 townships eligible for monitoring, the efficacy of an umbrella monitoring approach was assessed by calculating the percentage of townships that would be covered for each of the non-target species (at each level of decline detection) while monitoring for American marten at each of the three management objectives (10%, 25%, and 50% decline detection). The dplyr package in Program R was used for all data organization described above.

## Supplementary Information

Supplementary Information.

## Data Availability

The datasets generated and analyzed during the current study are available in the Figshare repository, https://figshare.com/s/355a185753036b1e6229.
