# Author Correction: Umbrella effect of monitoring protocols for mammals in the Northeast US

**DOI:** 10.1038/s41598-025-90252-4

**Published:** 2025-02-26

**Authors:** Alessio Mortelliti, Allison M. Brehm, Bryn E. Evans

**Affiliations:** Department of Wildlife, Fisheries, and Conservation Biology, University of Maine, 5755 Nutting Hall, Orono, ME 04469 USA

Correction to: *Scientific Reports* 10.1038/s41598-022-05791-x, published online 03 February 2022

The original version of this article contained an error in the formula that was used to calculate the sampling effort in Supplementary Information file. As the result of this error, the maps (S3a)–(S3n) and Supplementary Figure A1.1.–A1.3. were incorrect.

This error has now been corrected in the Supplementary Information file that accompanies the original Article.

## Supplementary Information

Supplementary Information.

