# Supplementary material for: Umbrella effect of monitoring protocols for mammals in the Northeast US
Source: Sci Rep. 2022 Feb 3;12:1893. doi: 10.1038/s41598-022-05791-x (PMC8814175; doi:10.1038/s41598-022-05791-x)
Supplement: Supplementary file 1 — Supplementary Information. [file 41598_2022_5791_MOESM1_ESM.pdf]

Umbrella effect of monitoring protocols for mammals in the Northeast US

Mortelliti* Alessio, Brehm Allison M., Evans Bryn E.

Supplemental Information

Page 2 – Figure S1: Maps and boxplots displaying the balanced study design implemented across the northern two-thirds of Maine, USA

Pages 3-14 – Figure S2: Plots of the occupancy modeling results for multi-season models of 12 species of terrestrial mammal in Maine, USA

Pages 15-28 – Figure S3: Maps of the survey effort required to detect 25% population declines for 14 species of terrestrial mammal in Maine, USA

Pages 29-35 – Appendix S1: Case study of the effectiveness of an umbrella monitoring protocol prioritizing first American marten, and second coyote

Pages 36-40 – Appendix S2: Example trail camera images of the 14 mammalian species reported on

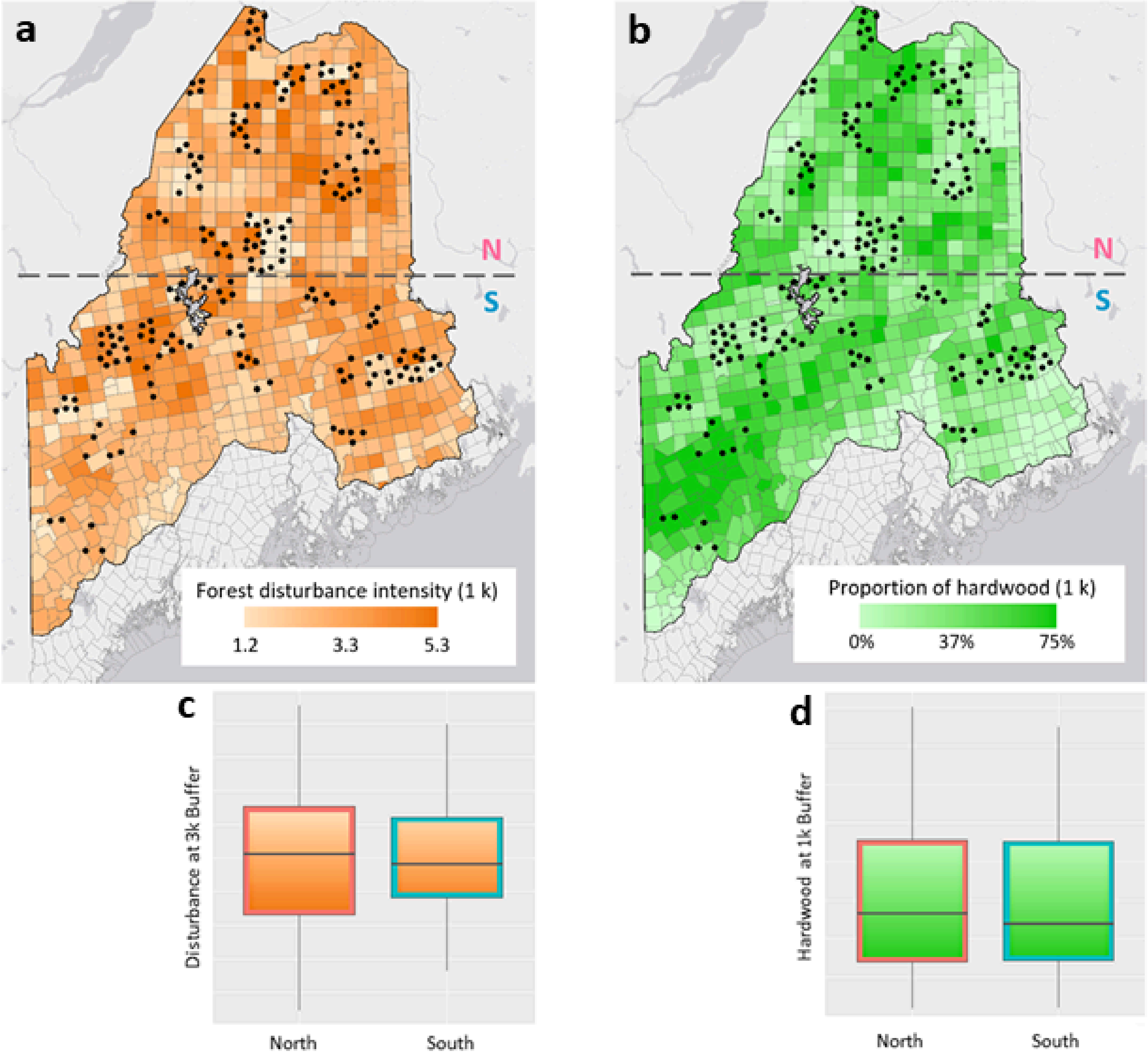

Fig. S1: Balanced study design across varying degrees of (**a**) forest disturbance intensity and (**b**) proportion of hardwood species present. Black dots show the location of camera trap sites, which were strategically placed to balance the survey effort in both north and south of the study area (735 townships in north and central Maine, USA). Testing for correlation indicated that the latitude at which survey sites were placed was not predictive of either the forest disturbance (**c**) or the hardwood (**d**) characteristics.

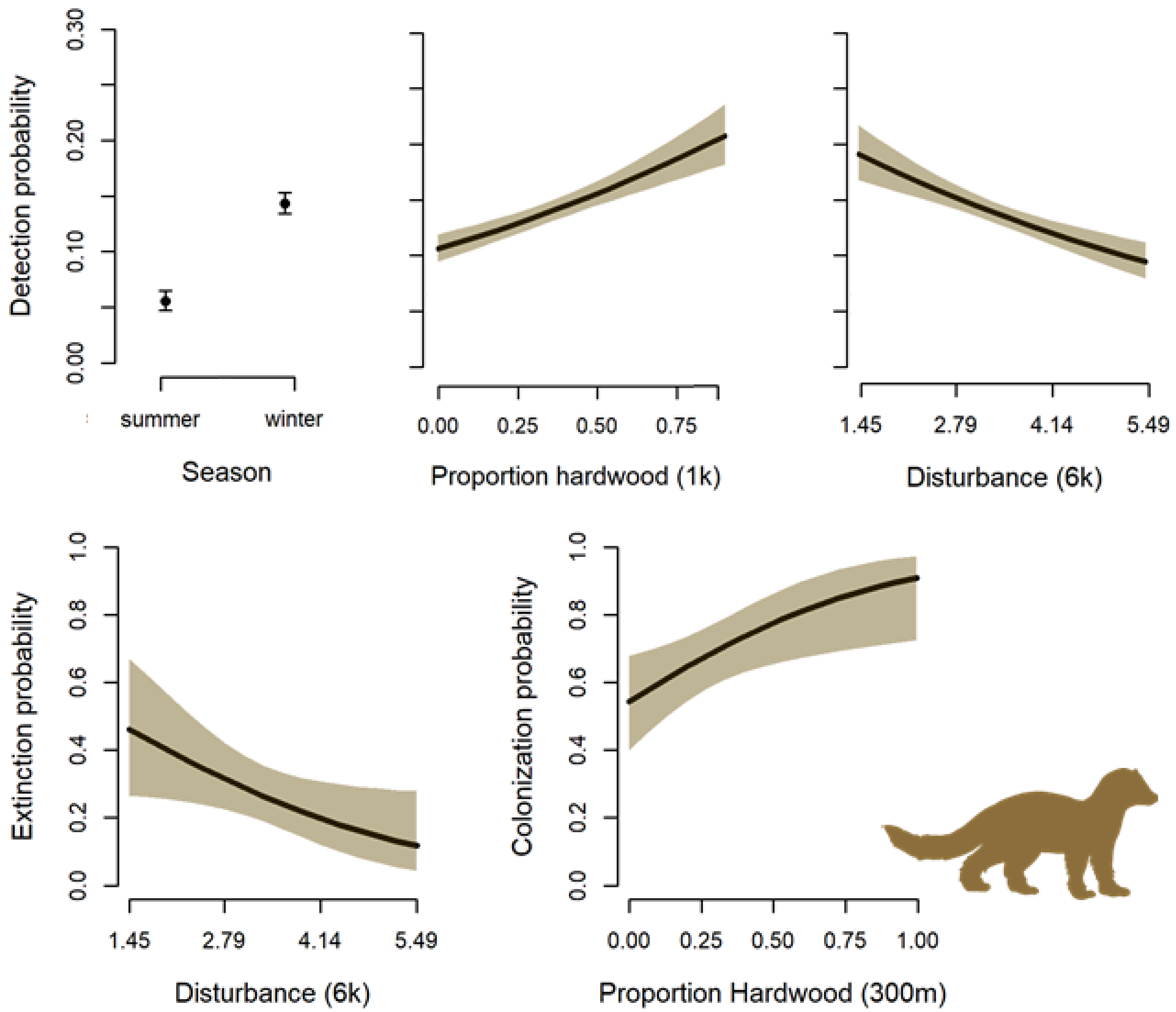

Fig. S2a) Results for fisher occupancy models. Shown are the predictions from the top ranked multi-season occupancy models from 197 camera survey stations deployed in Maine, USA. The shaded area includes the 95% CI.

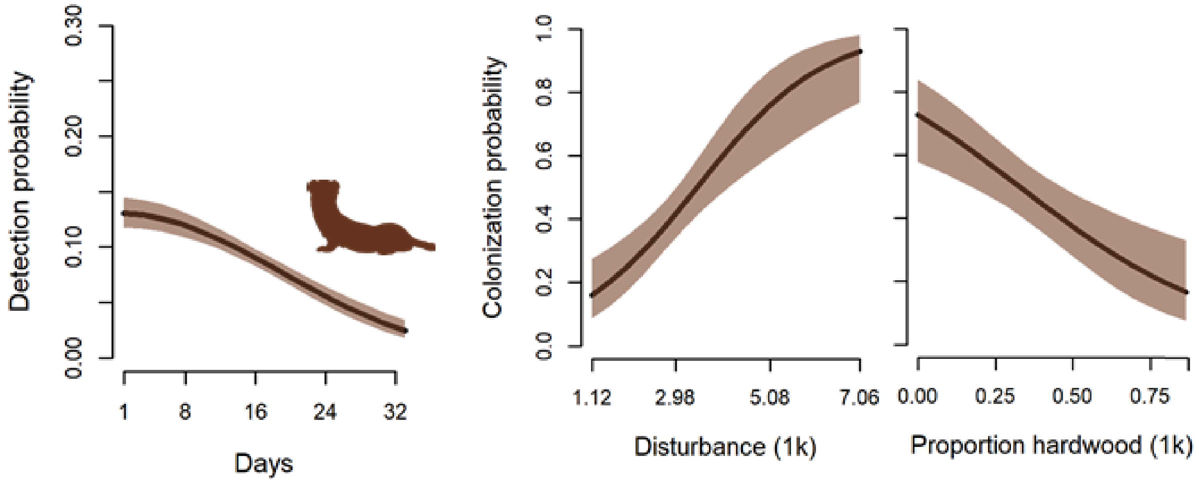

Fig. S2b) Results for short-tailed weasel occupancy models. Shown are the predictions from the top ranked multi-season occupancy models from 197 camera survey stations deployed in Maine, USA. The shaded area includes the 95% CI.

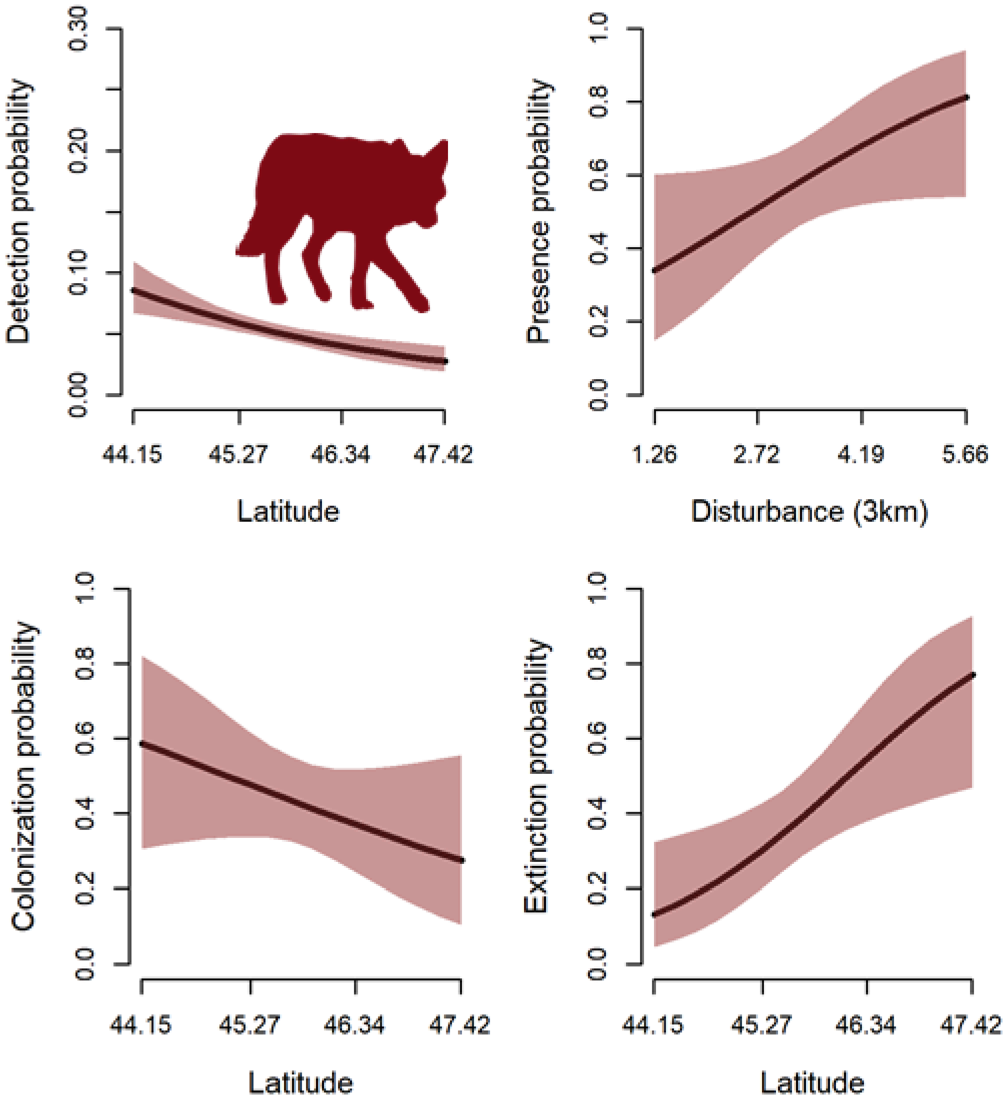

Fig. S2c) Results for coyote occupancy models. Shown are the predictions from the top ranked multi-season occupancy models from 197 camera survey stations deployed in Maine, USA. The shaded area includes the 95% CI.

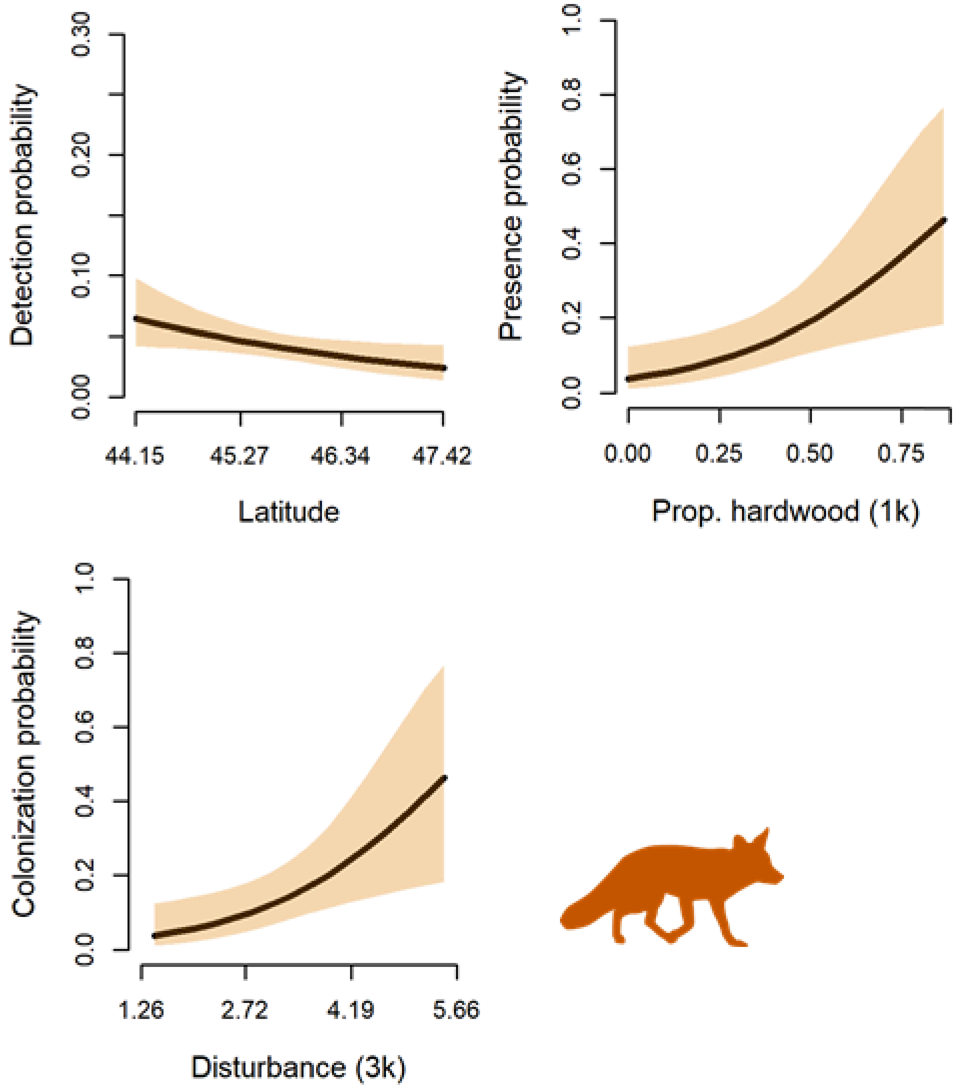

Fig. S2d) Results for red fox occupancy models. Shown are the predictions from the top ranked multi-season occupancy models from 197 camera survey stations deployed in Maine, USA. The shaded area includes the 95% CI.

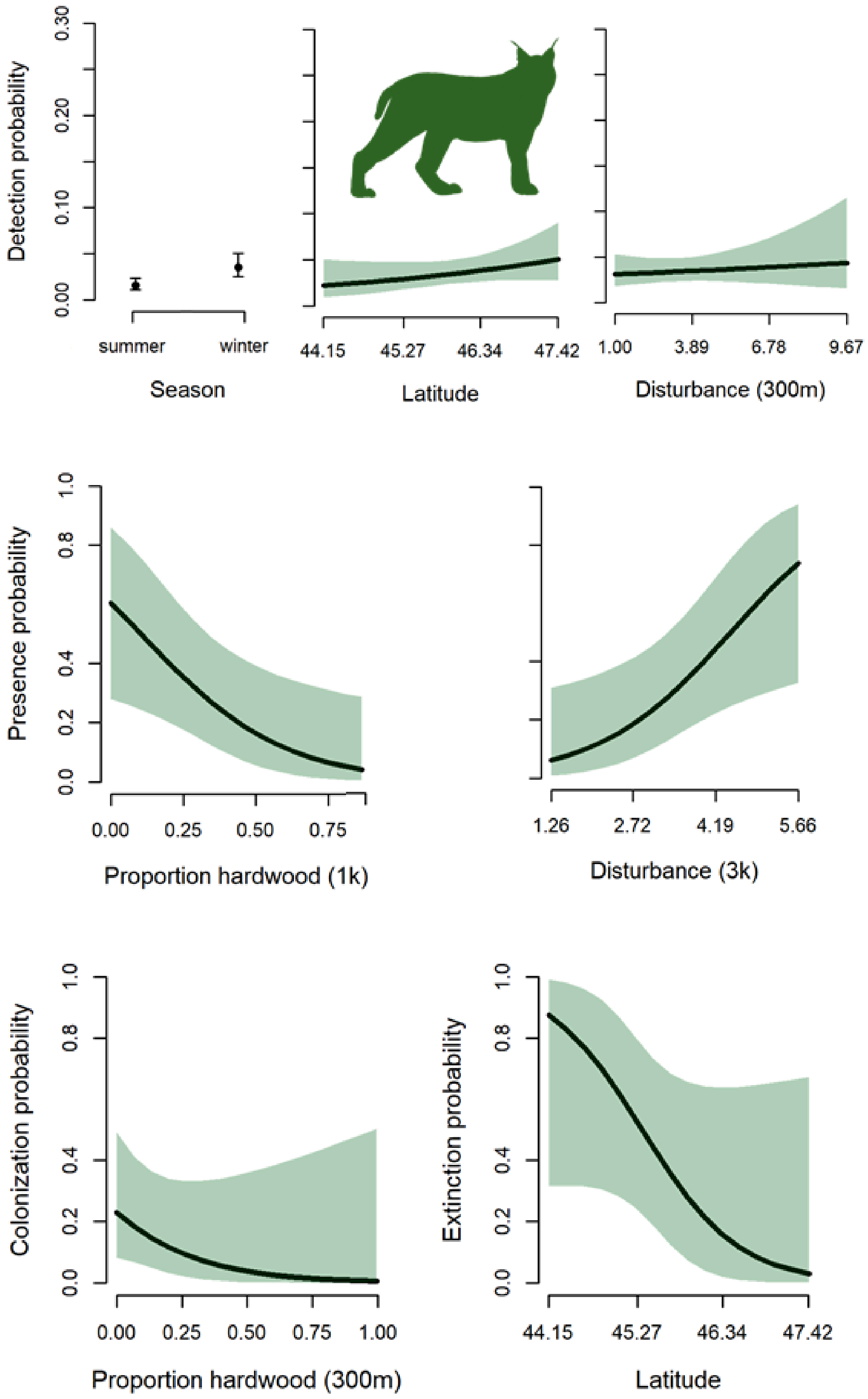

Fig S2e) Results for Canada lynx occupancy models. Shown are the predictions from the top ranked multi-season occupancy models from 197 camera survey stations deployed in Maine, USA. The shaded area includes the 95% CI.

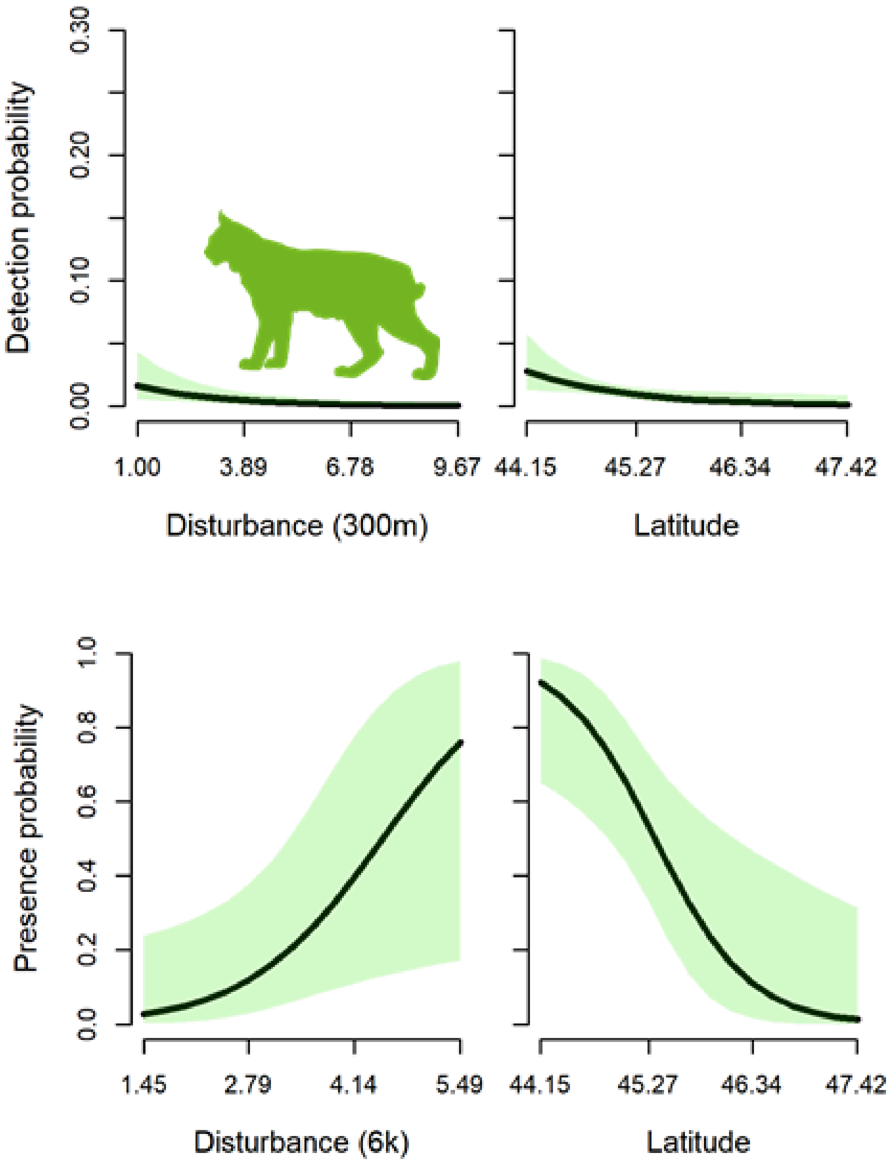

Fig S2f) Results for bobcat occupancy models. Shown are the predictions from the top ranked multi-season occupancy models from 197 camera survey stations deployed in Maine, USA. The shaded area includes the 95% CI.

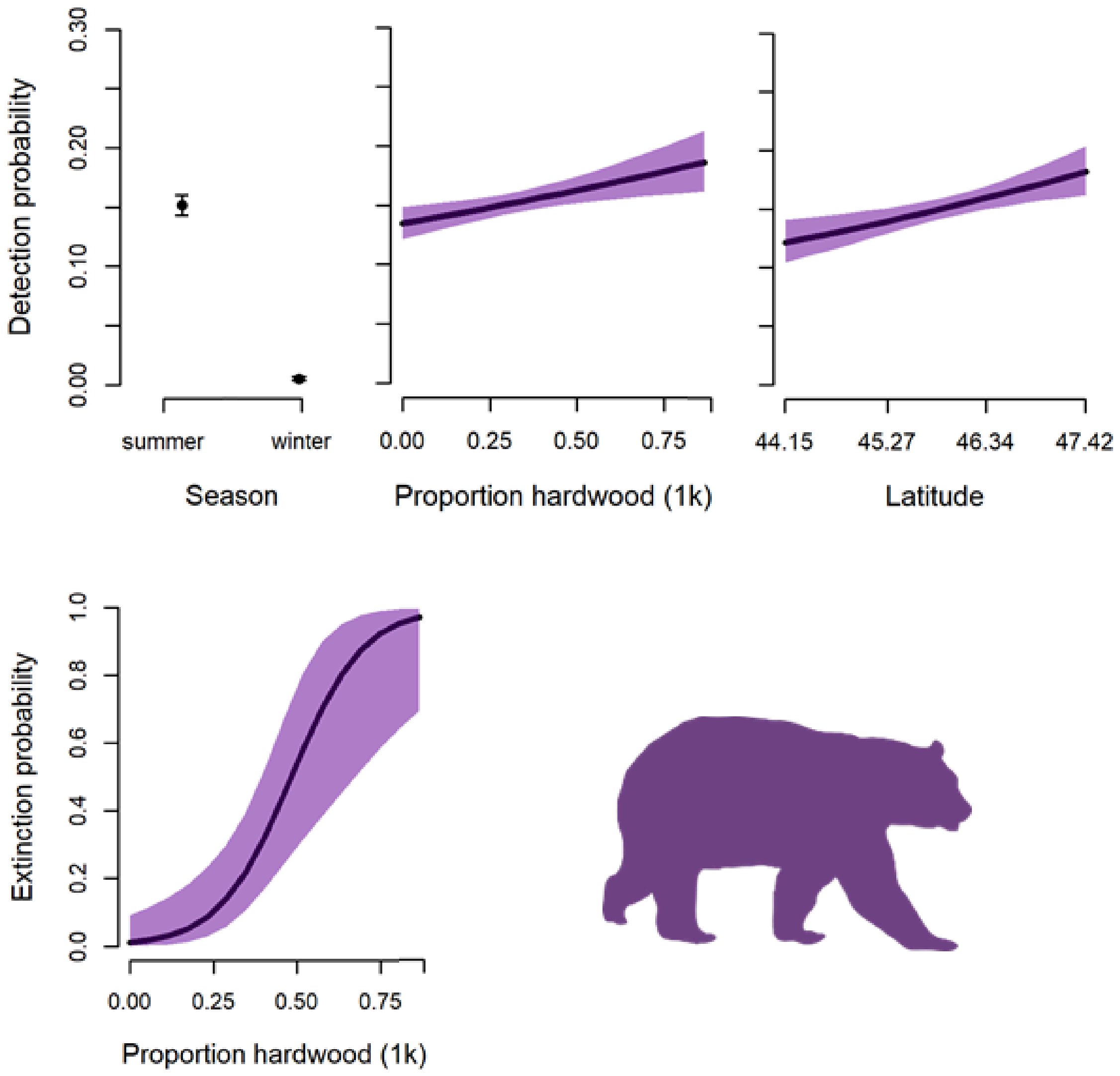

Fig S2g) Results for American black bear occupancy models. Shown are the predictions from the top ranked multi-season occupancy models from 197 camera survey stations deployed in Maine, USA. The shaded area includes the 95% CI.

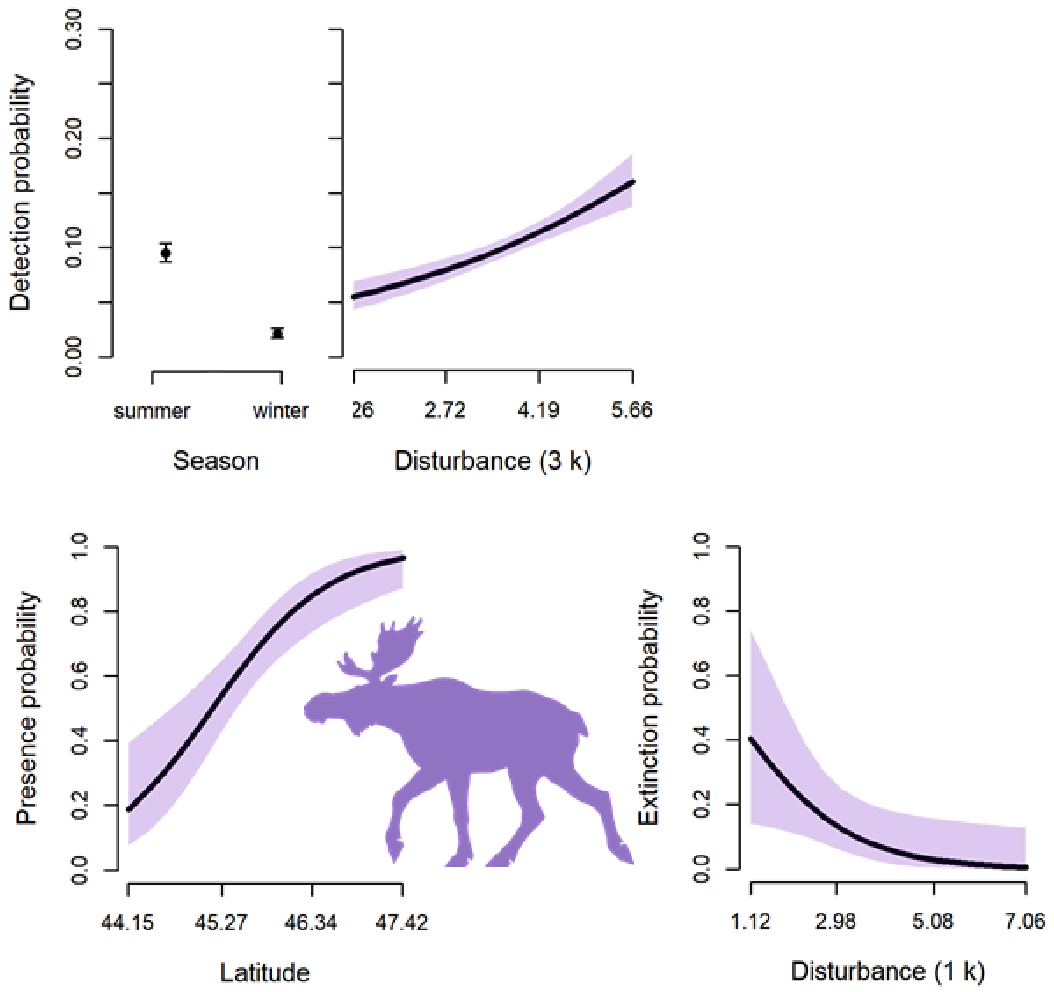

Fig. S2h) Results for moose occupancy models. Shown are the predictions from the top ranked multi-season occupancy models from 197 camera survey stations deployed in Maine, USA. The shaded area includes the 95% CI.

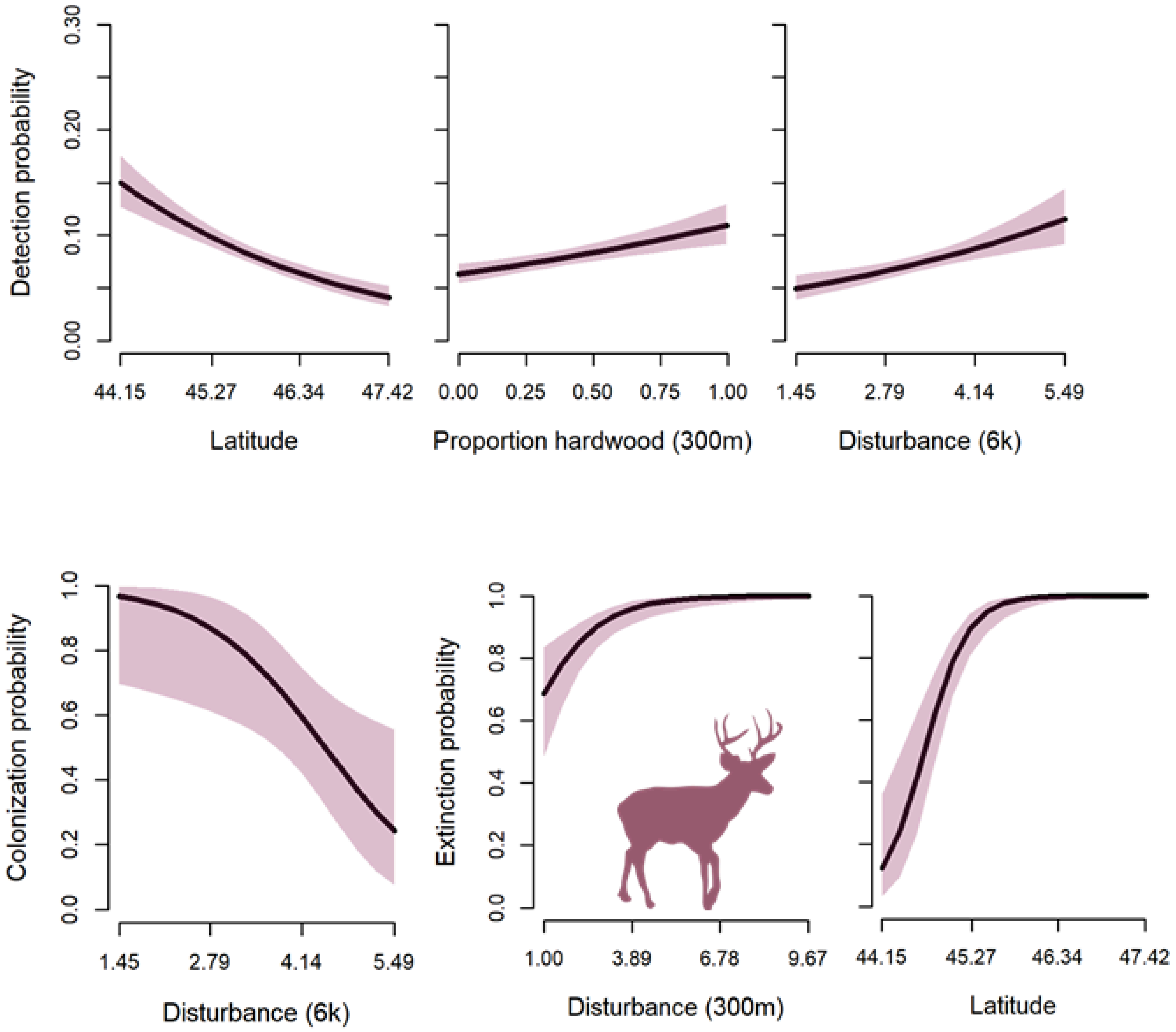

Fig. S2i) Results for white-tailed deer occupancy models. Shown are the predictions from the top ranked multi-season occupancy models from 197 camera survey stations deployed in Maine, USA. The shaded area includes the 95% CI.

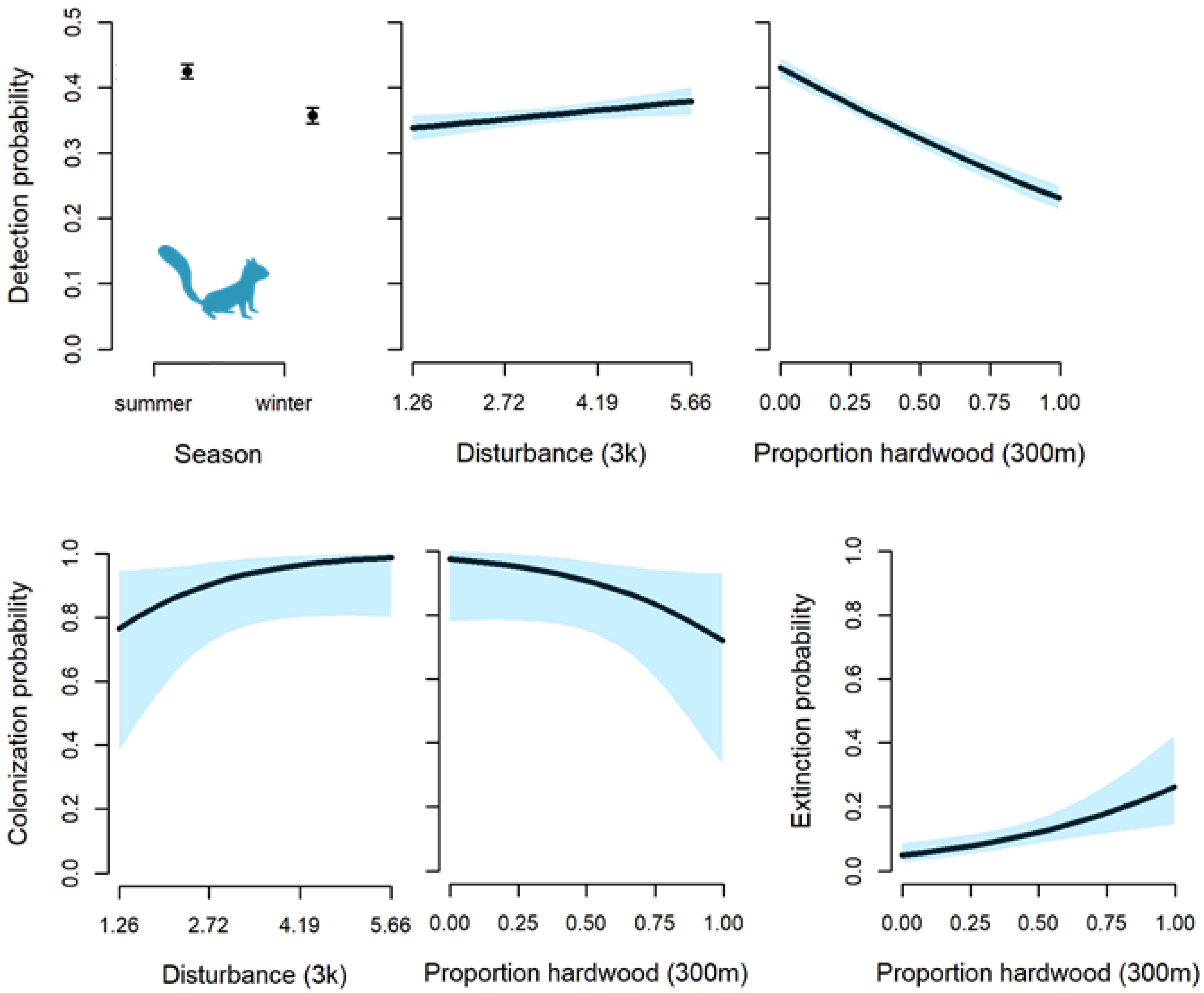

Fig. S2j) Results for American red squirrel occupancy models. Shown are the predictions from the top ranked multi-season occupancy models from 197 camera survey stations deployed in Maine, USA. The shaded area includes the 95% CI.

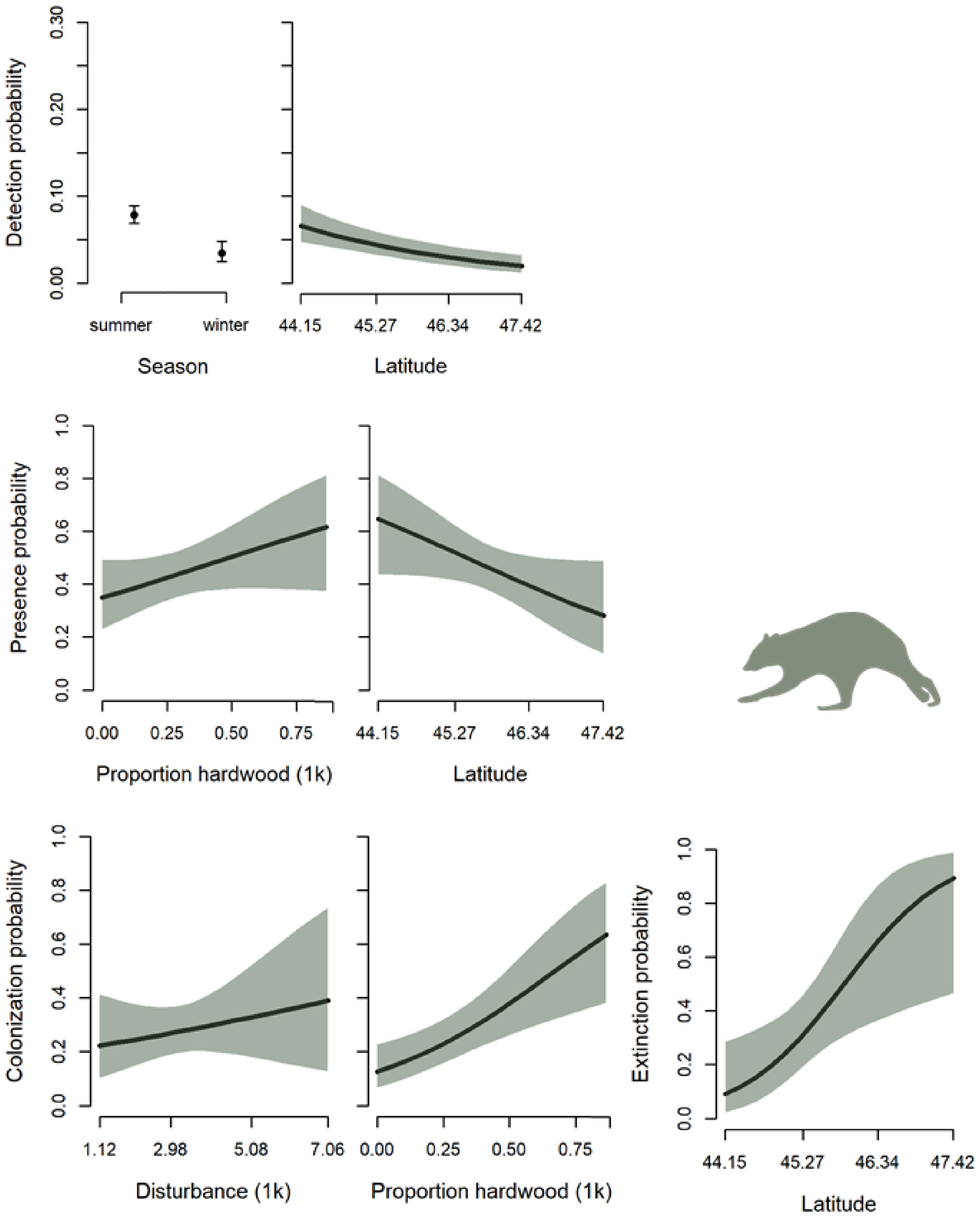

Fig. S2k) Results for raccoon occupancy models. Shown are the predictions from the top ranked multi-season occupancy models from 197 camera survey stations deployed in Maine, USA. The shaded area includes the 95% CI.

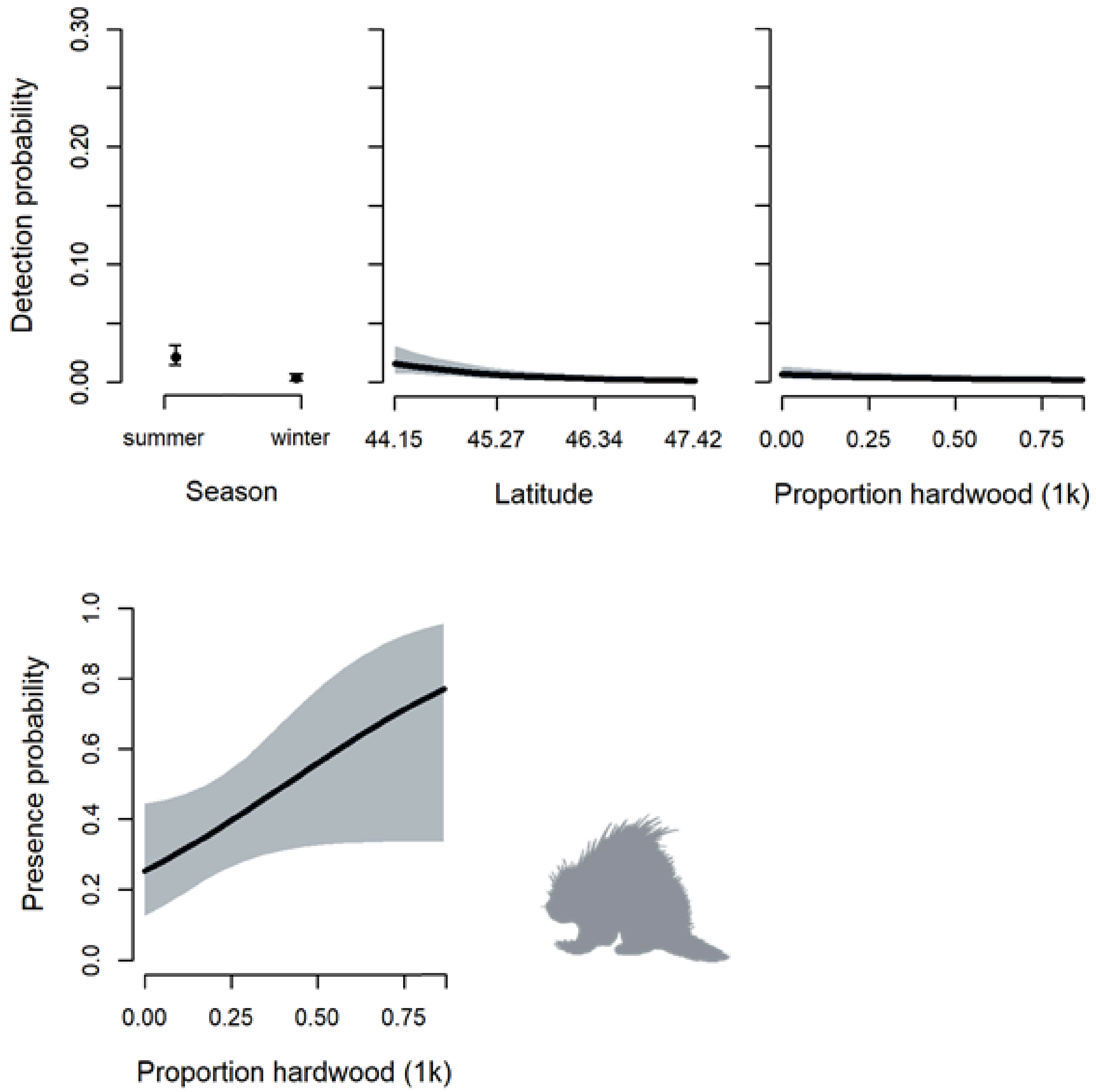

Fig. S2l) Results for North American porcupine occupancy models. Shown are the predictions from the top ranked multi-season occupancy models from 197 camera survey stations deployed in Maine, USA. The shaded area includes the 95% CI.

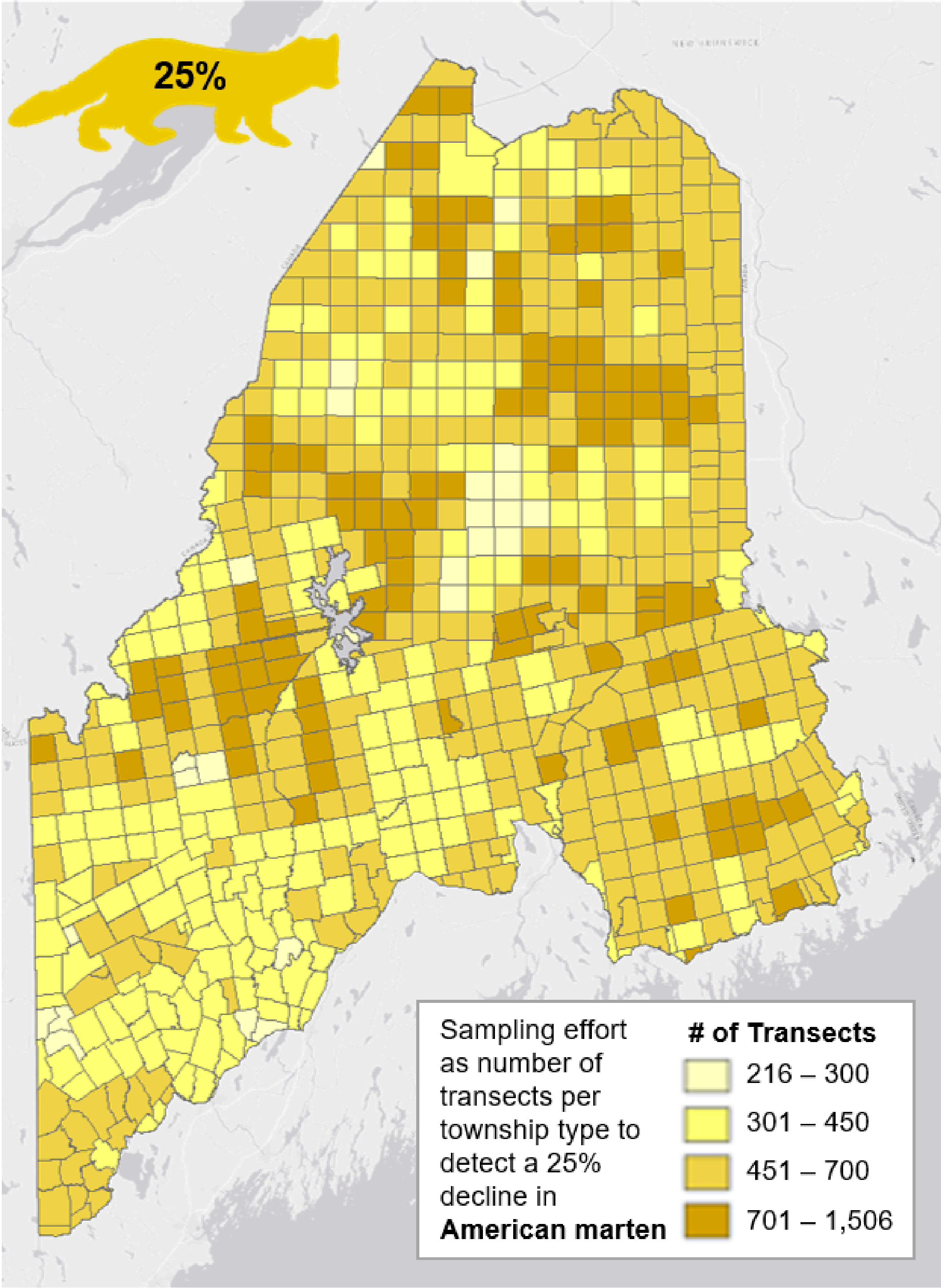

Fig. S3a) Survey effort for American marten in terms of the number of transects to be deployed across four categories of townships in Maine, USA. Sampling effort refers to the total number of transects to be deployed across all townships of the same category, not per township. As an example, to detect a 25% decline of marten across all areas colored in lightest yellow, 300 transects are required.

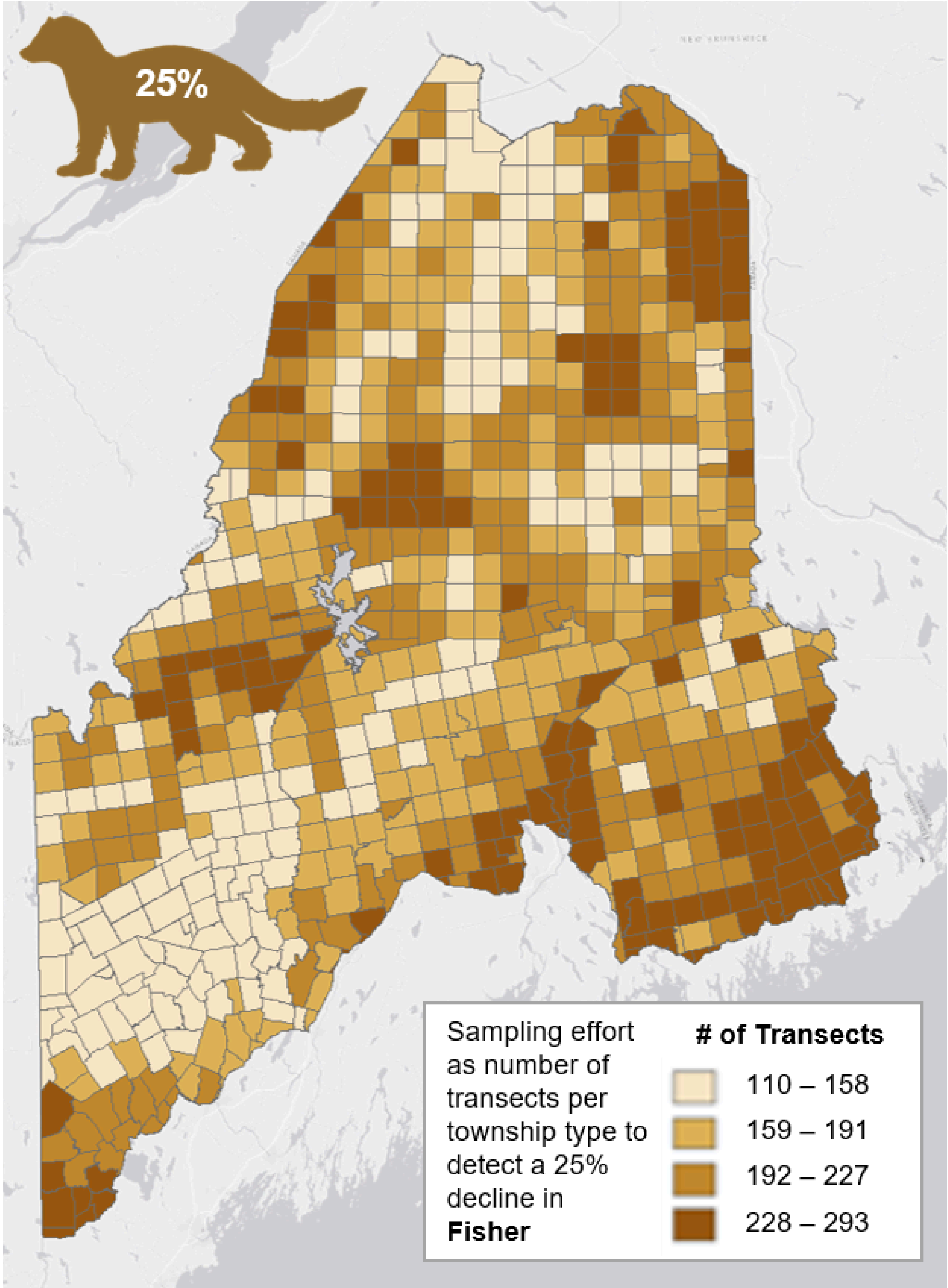

Fig. S3b) Survey effort for fisher in terms of the number of transects to be deployed across four categories of townships in Maine, USA. Sampling effort refers to the total number of transects to be deployed across all townships of the same category, not per township. As an example, to detect a 25% decline of fisher across all areas colored in lightest tan, 158 transects are required.

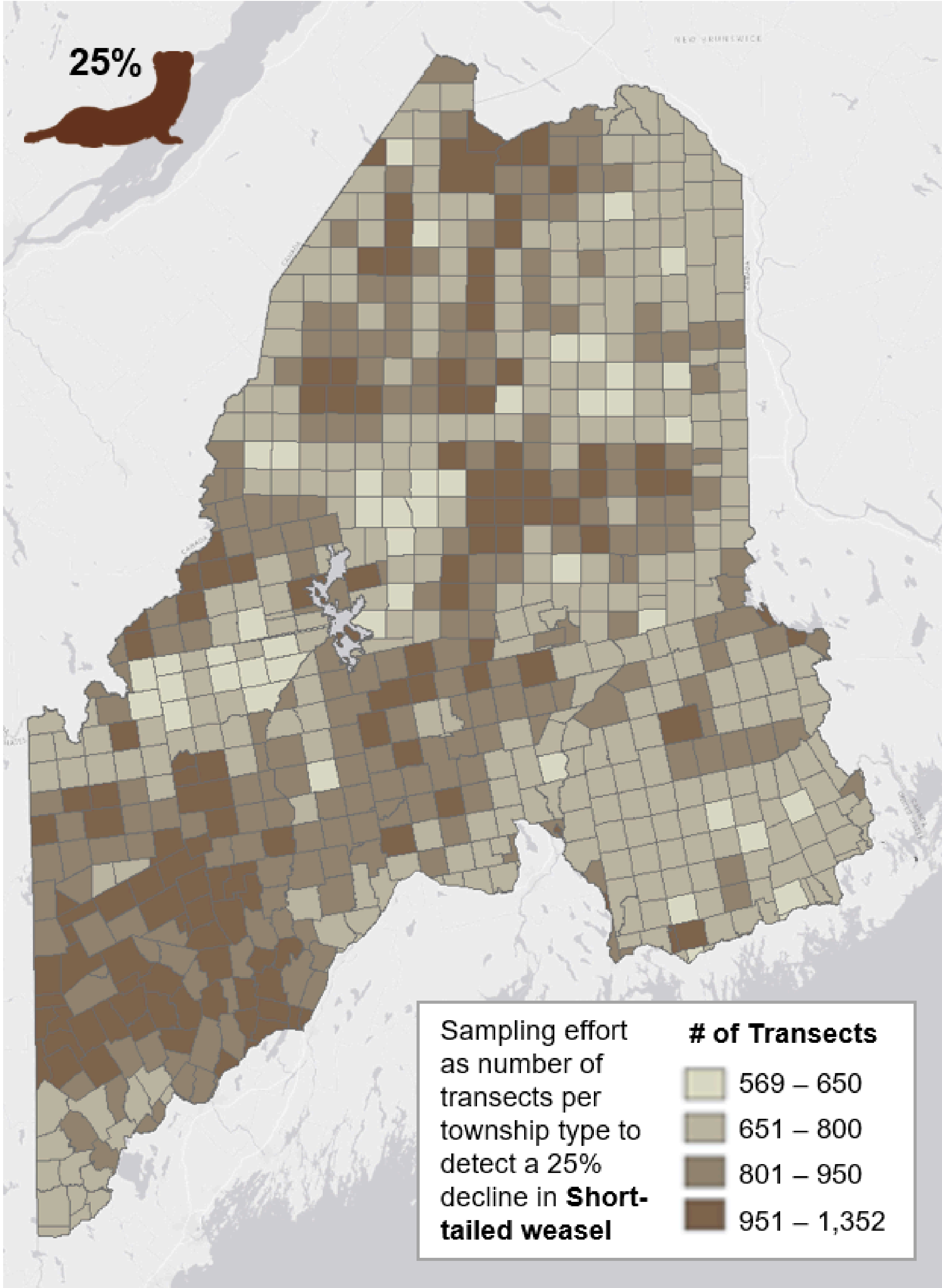

Fig. S3c) Survey effort for short-tailed weasel in terms of the number of transects to be deployed across four categories of townships in Maine, USA. Sampling effort refers to the total number of transects to be deployed across all townships of the same category, not per township. As an example, to detect a 25% decline of weasel across all areas colored in lightest brown, 650 transects are required.

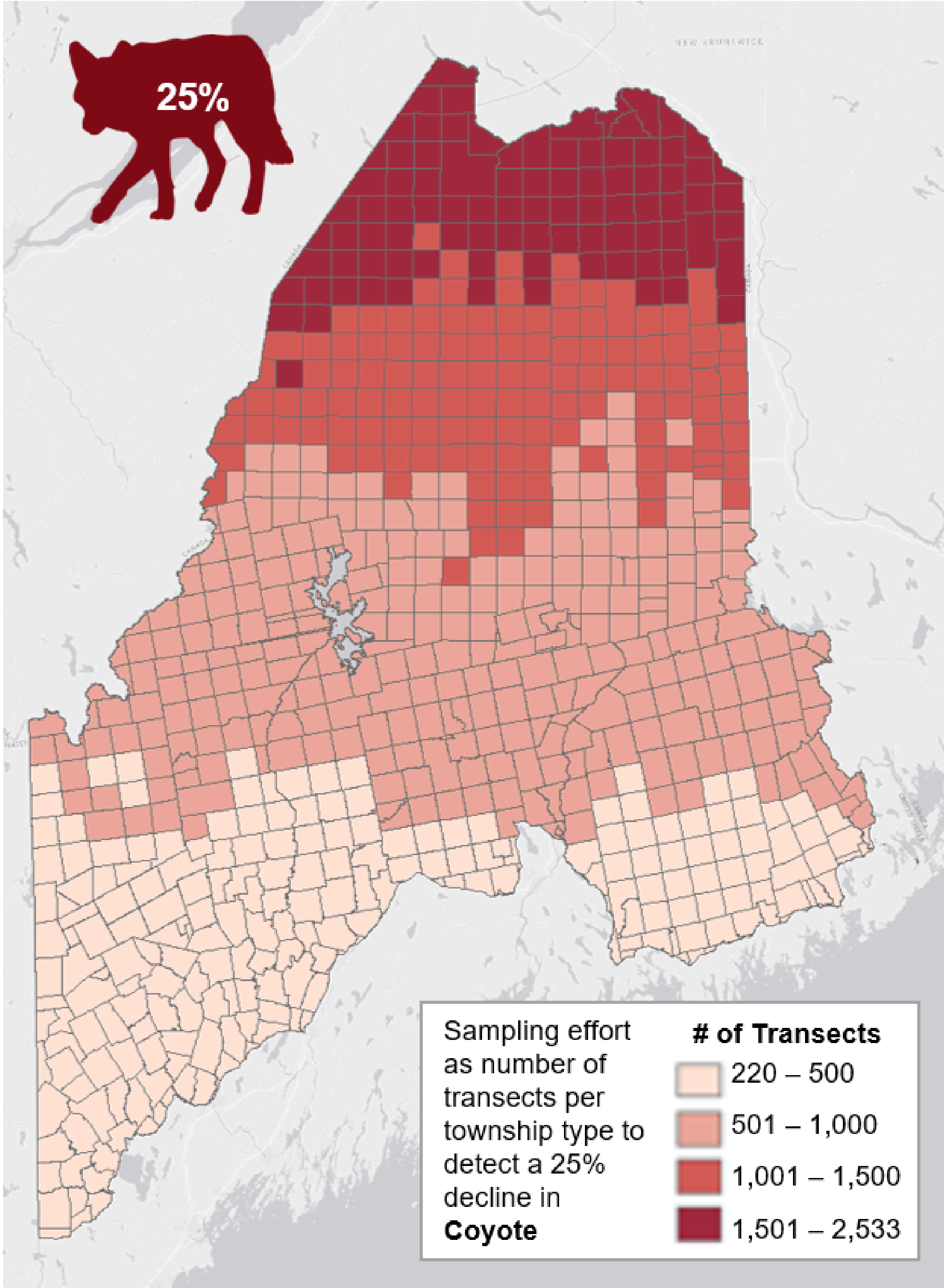

Fig. S3d) Survey effort for coyote in terms of the number of transects to be deployed across four categories of townships in Maine, USA. Sampling effort refers to the total number of transects to be deployed across all townships of the same category, not per township. As an example, to detect a 25% decline of coyote across all areas colored in lightest red, 500 transects are required.

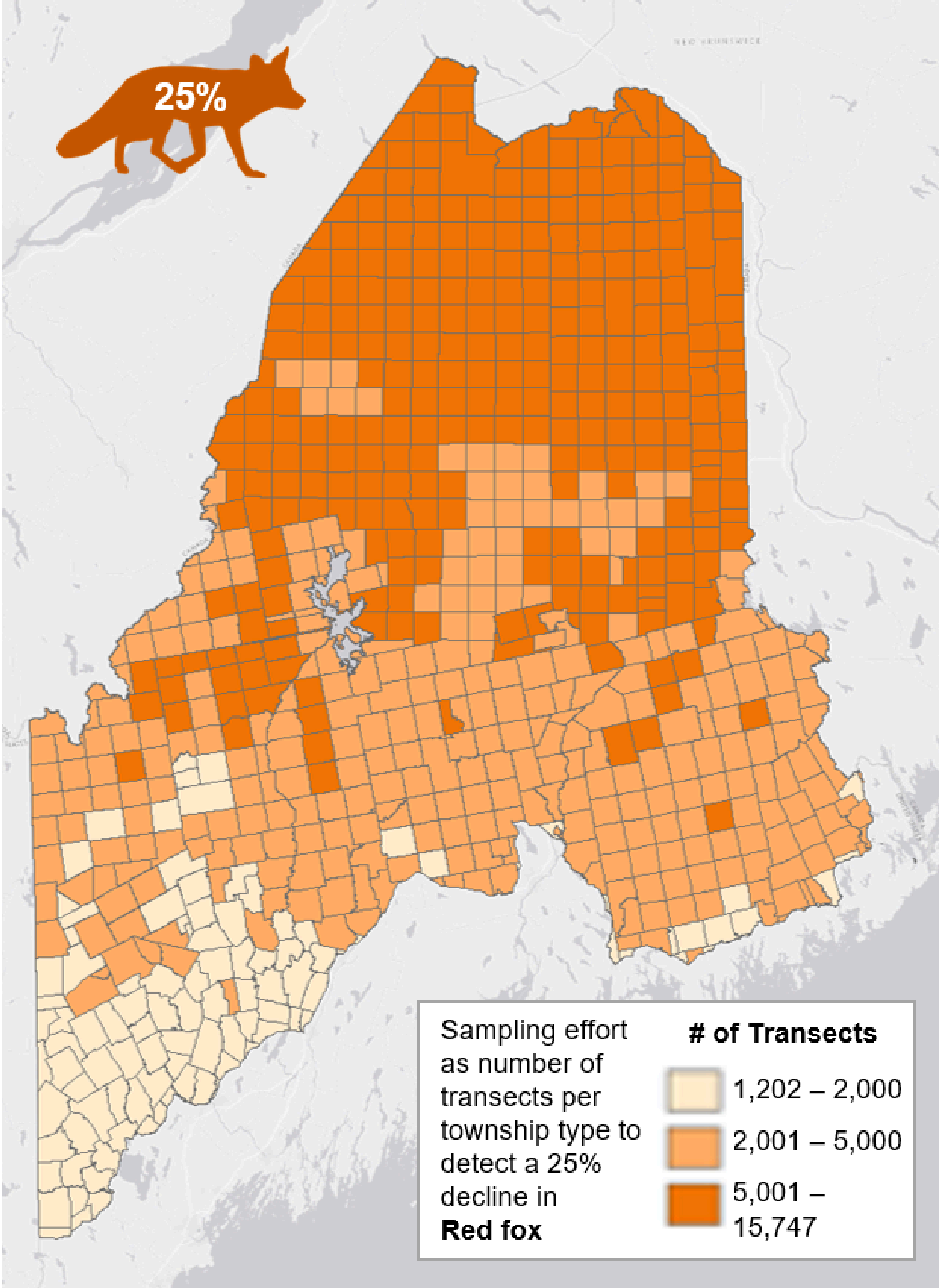

Fig. S3e) Survey effort for red fox in terms of the number of transects to be deployed across four categories of townships in Maine, USA. Sampling effort refers to the total number of transects to be deployed across all townships of the same category, not per township. As an example, to detect a 25% decline of red fox across all areas colored in lightest orange, 2,000 transects are required.

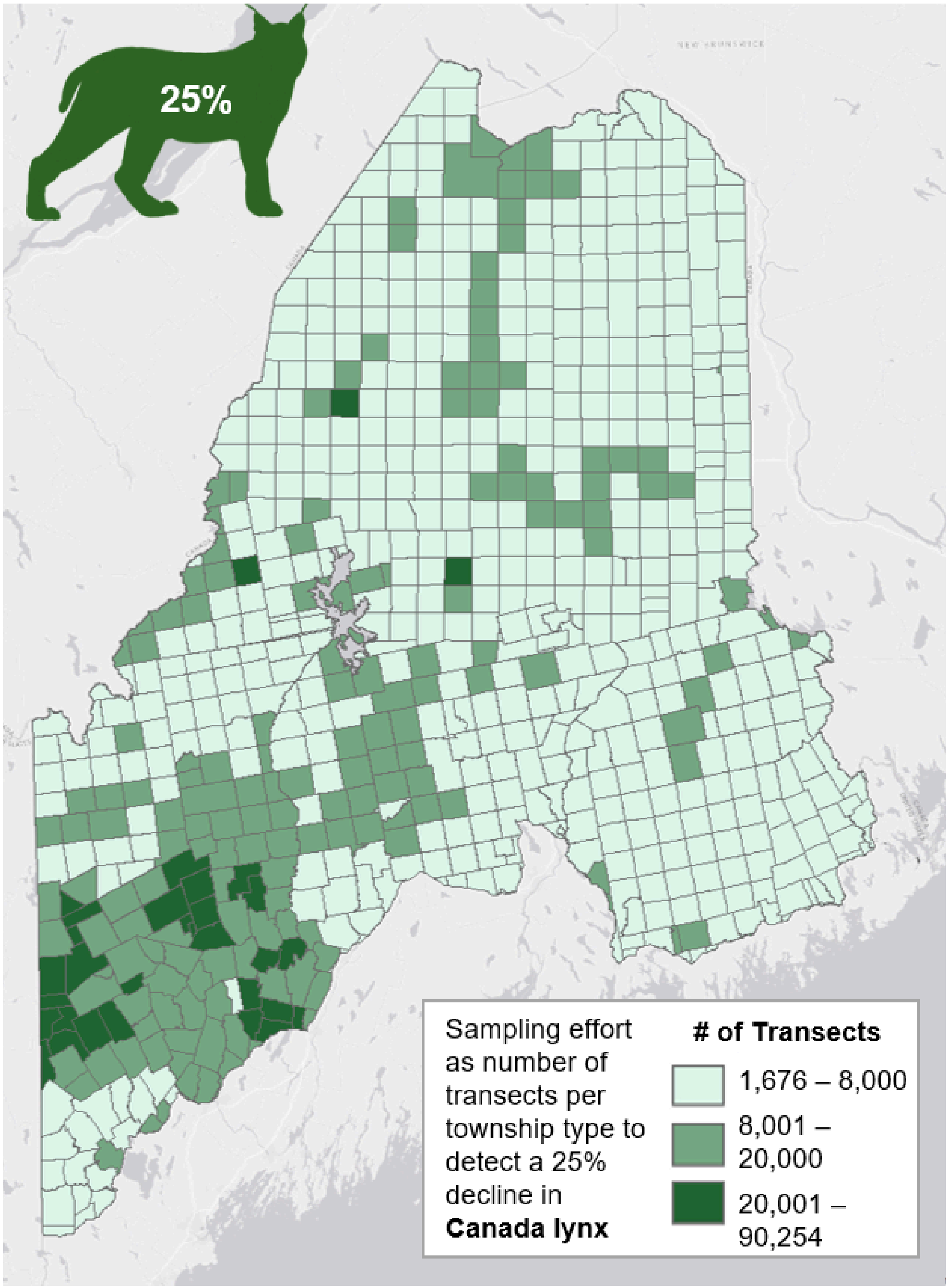

Fig. S3f) Survey effort for Canada lynx in terms of the number of transects to be deployed across four categories of townships in Maine, USA. Sampling effort refers to the total number of transects to be deployed across all townships of the same category, not per township. As an example, to detect a 25% decline of lynx across all areas colored in lightest green, 8,000 transects are required.

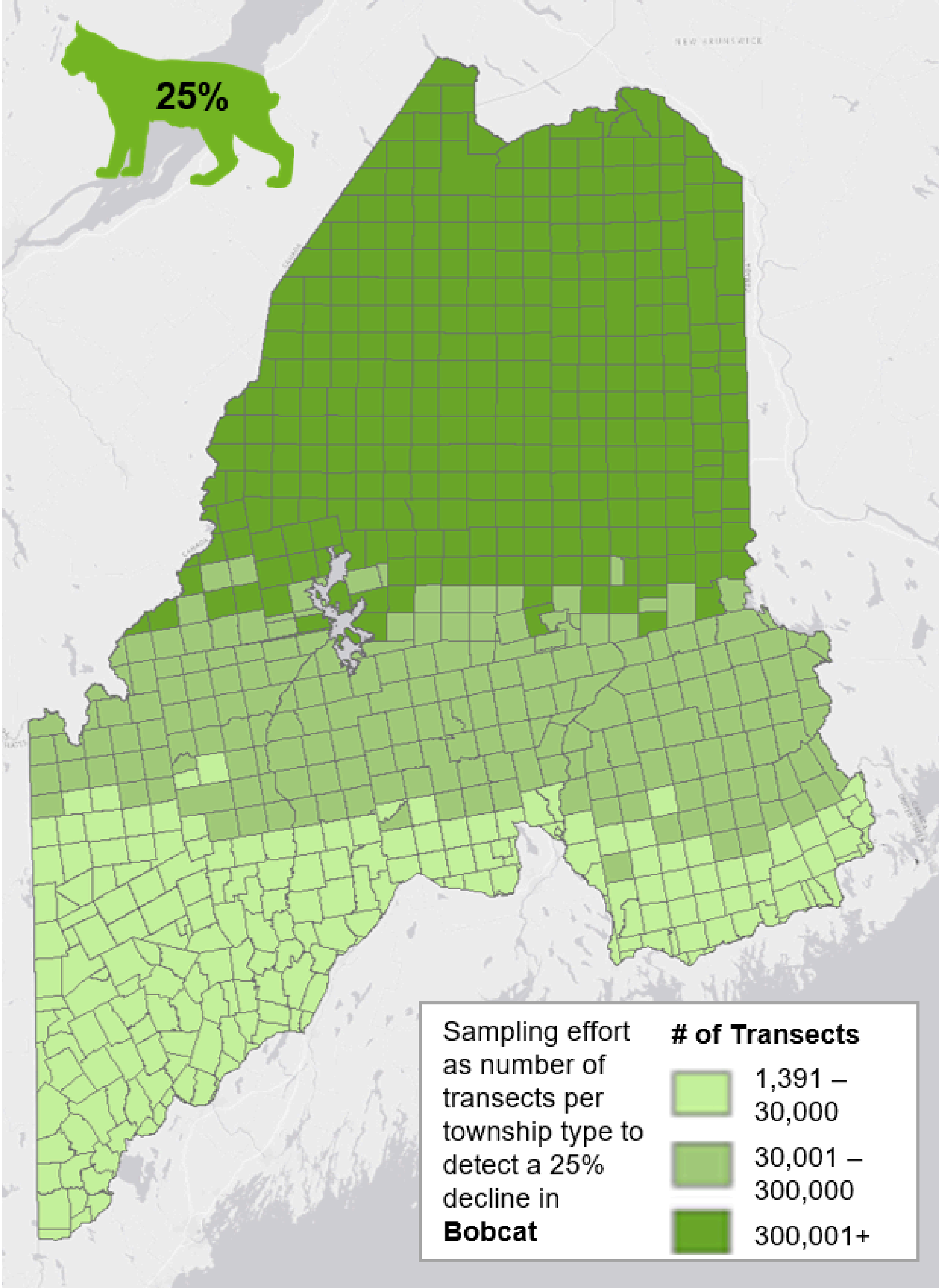

Fig. S3g) Survey effort for bobcat in terms of the number of transects to be deployed across four categories of townships in Maine, USA. Sampling effort refers to the total number of transects to be deployed across all townships of the same category, not per township. As an example, to detect a 25% decline of bobcat across all areas colored in lightest neon green, 30,000 transects are required.

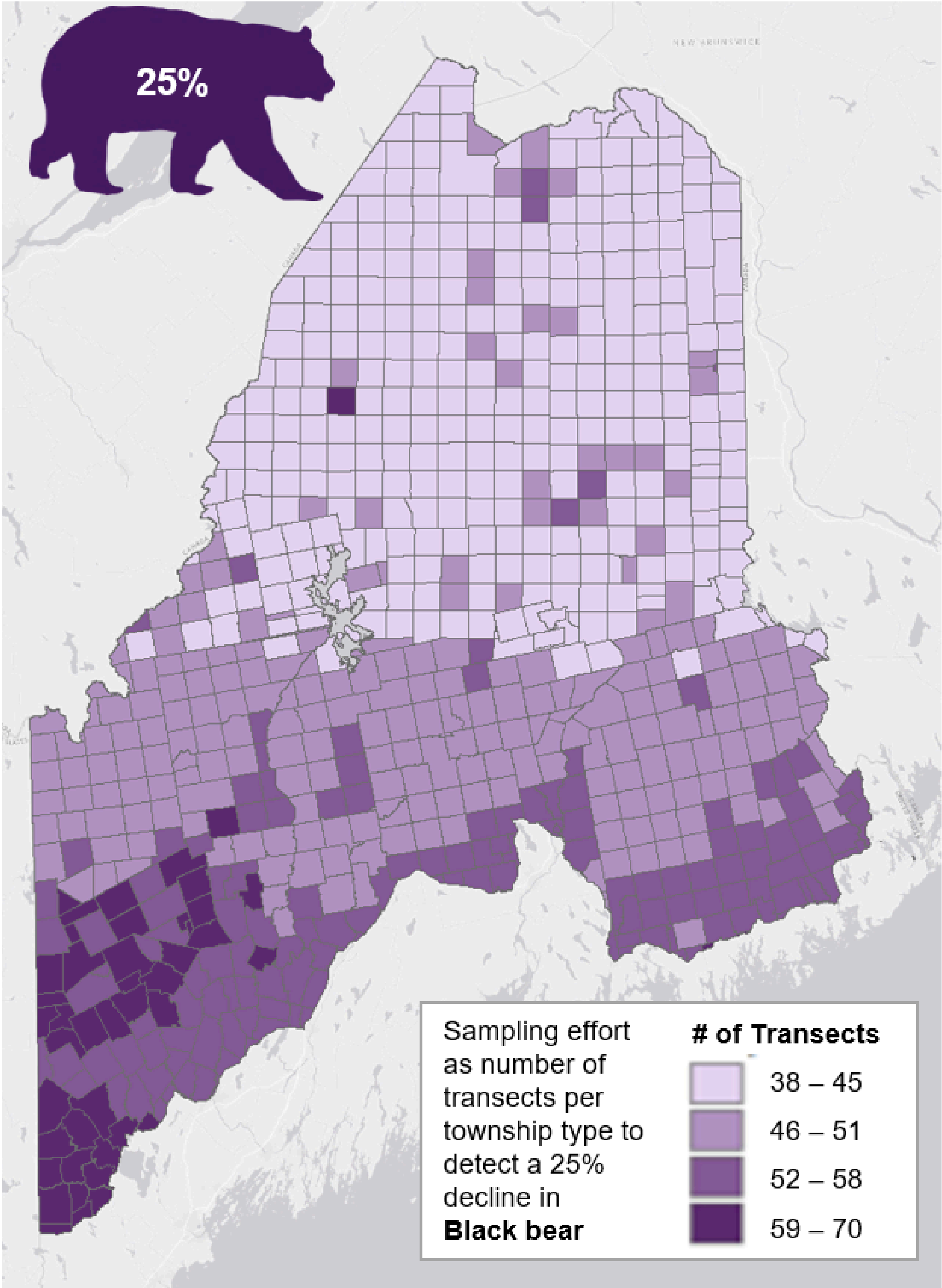

Fig. S3h) Survey effort for black bear in terms of the number of transects to be deployed across three categories of townships in Maine, USA. Sampling effort refers to the total number of transects to be deployed across all townships of the same category, not per township. Given the high occupancy estimates across the range, minimum survey effort is low (for example, 45 transects to detect a 25% decline, shown in the lightest purple), but we underscore the need to follow basic sampling design rules, such as making sure that the areas sampled are representative and biologically meaningful.

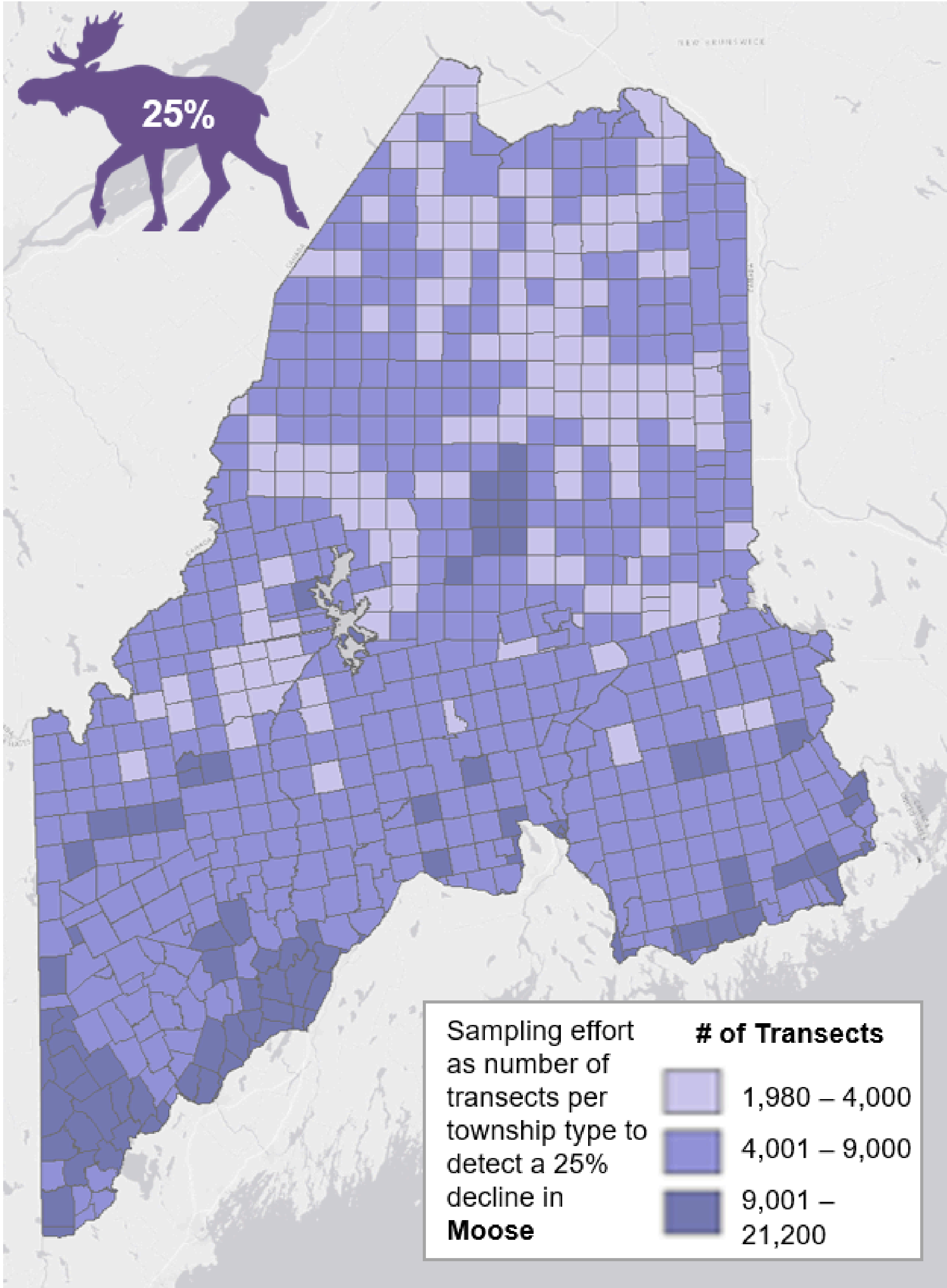

Fig. S3i) Survey effort for moose in terms of the number of transects to be deployed across four categories of townships in Maine, USA. Sampling effort refers to the total number of transects to be deployed across all townships of the same category, not per township. As an example, to detect a 25% decline of moose across all areas colored in lightest lavender, 4,000 transects are required.

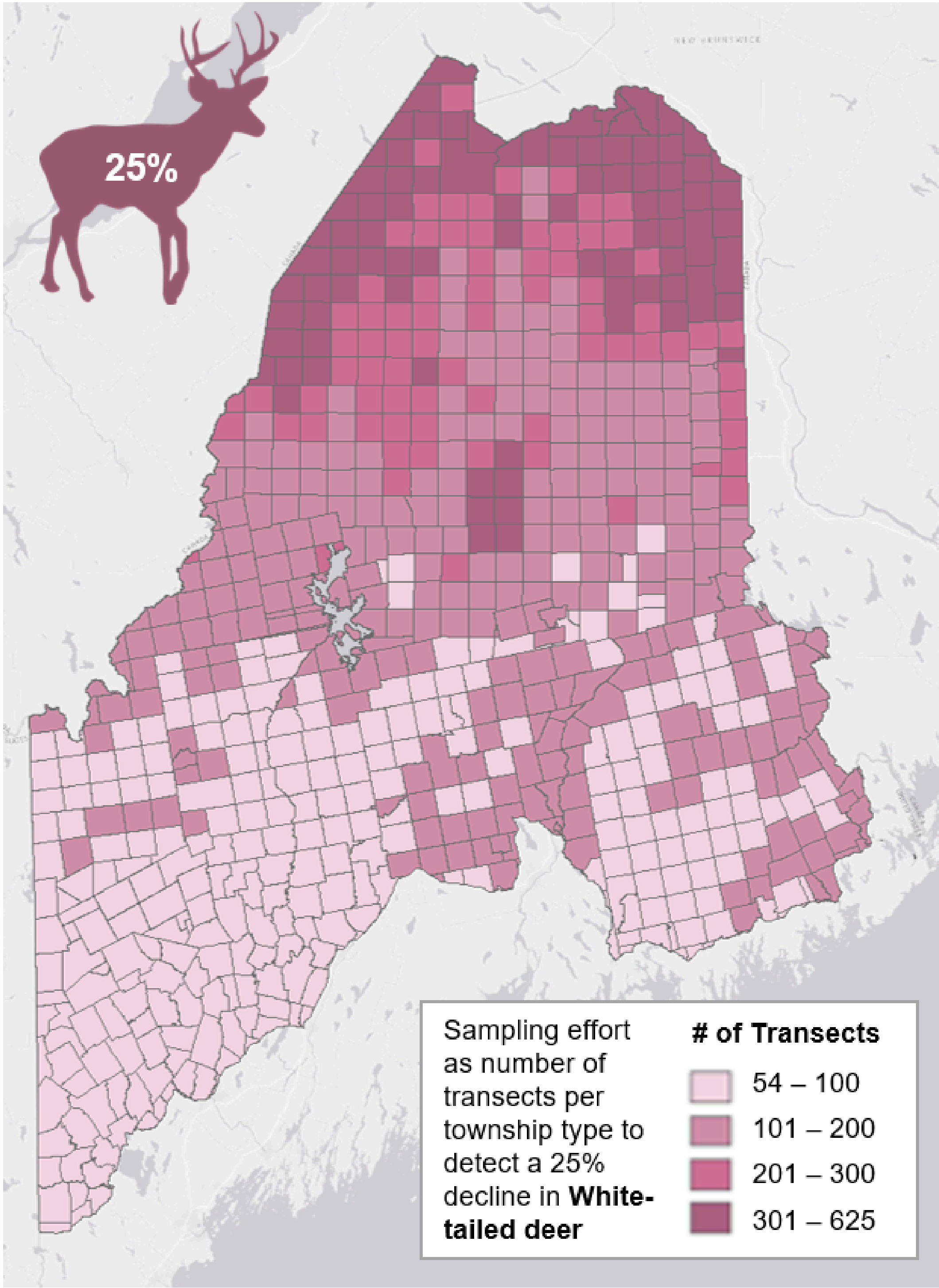

Fig. S3j) Survey effort for white-tailed deer in terms of the number of transects to be deployed across four categories of townships in Maine, USA. Sampling effort refers to the total number of transects to be deployed across all townships of the same category, not per township. As an example, to detect a 25% decline of white-tailed deer across all areas colored in lightest pink, 100 transects are required.

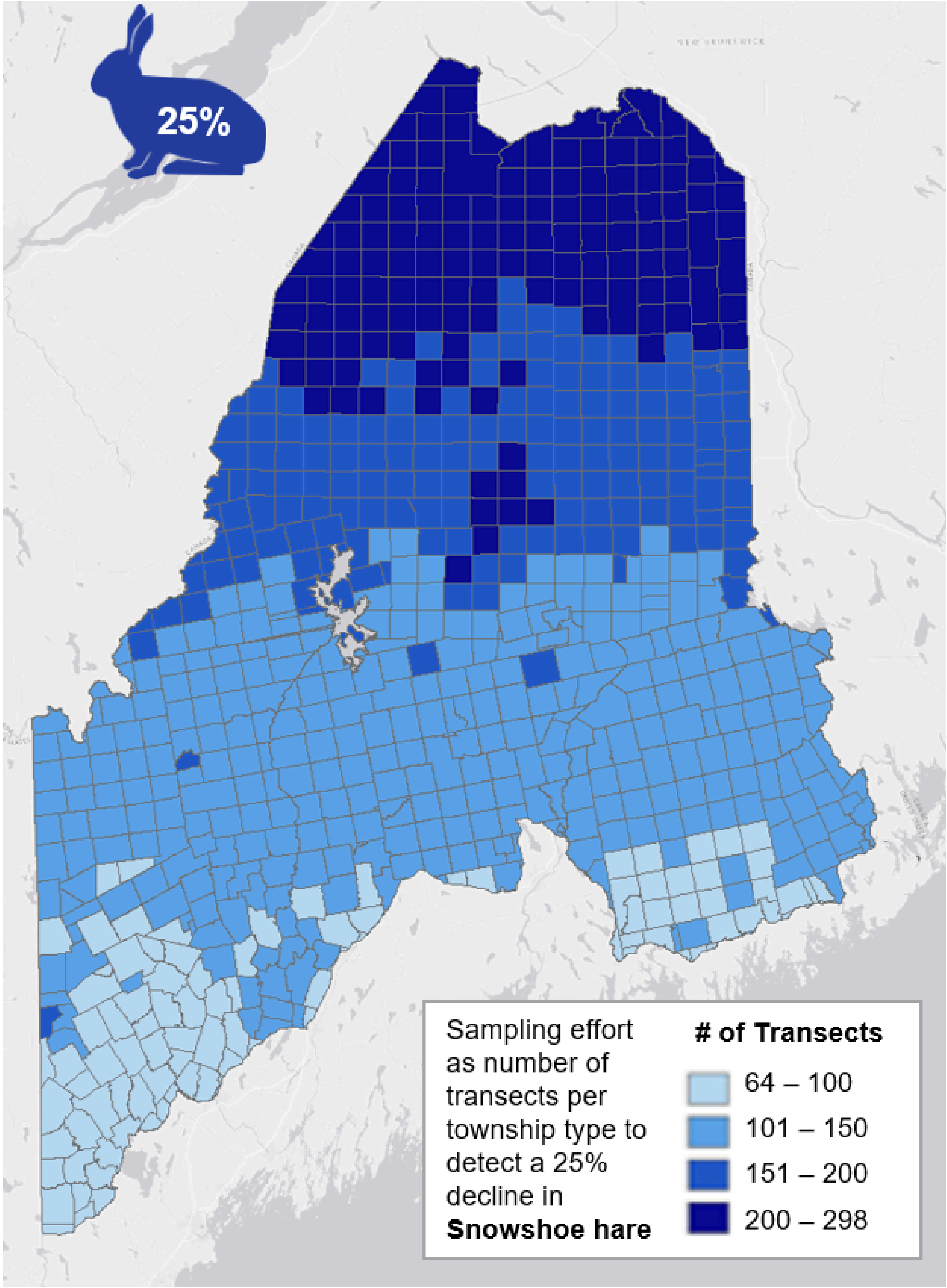

Fig. S3k) Survey effort for snowshoe hare in terms of the number of transects to be deployed across four categories of townships in Maine, USA. Sampling effort refers to the total number of transects to be deployed across all townships of the same category, not per township. As an example, to detect a 25% decline of snowshoe hare across all areas colored in lightest blue, 100 transects are required.

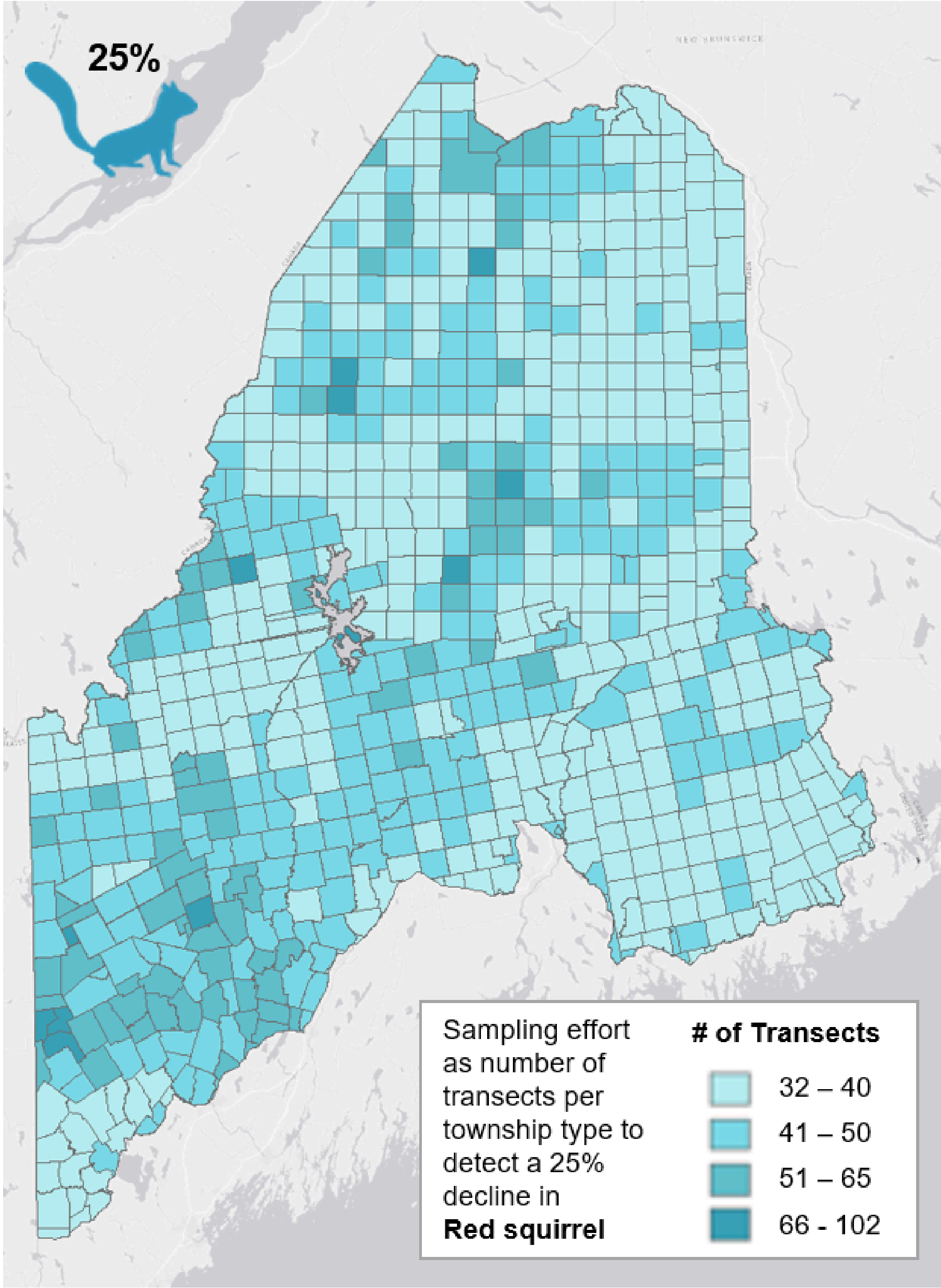

Fig S3l) Survey effort for red squirrel in terms of the number of transects to be deployed across four categories of townships in Maine, USA. Sampling effort refers to the total number of transects to be deployed across all townships of the same category, not per township. As an example, to detect a 25% decline of red squirrel across all areas colored in lightest teal, 40 transects are required.

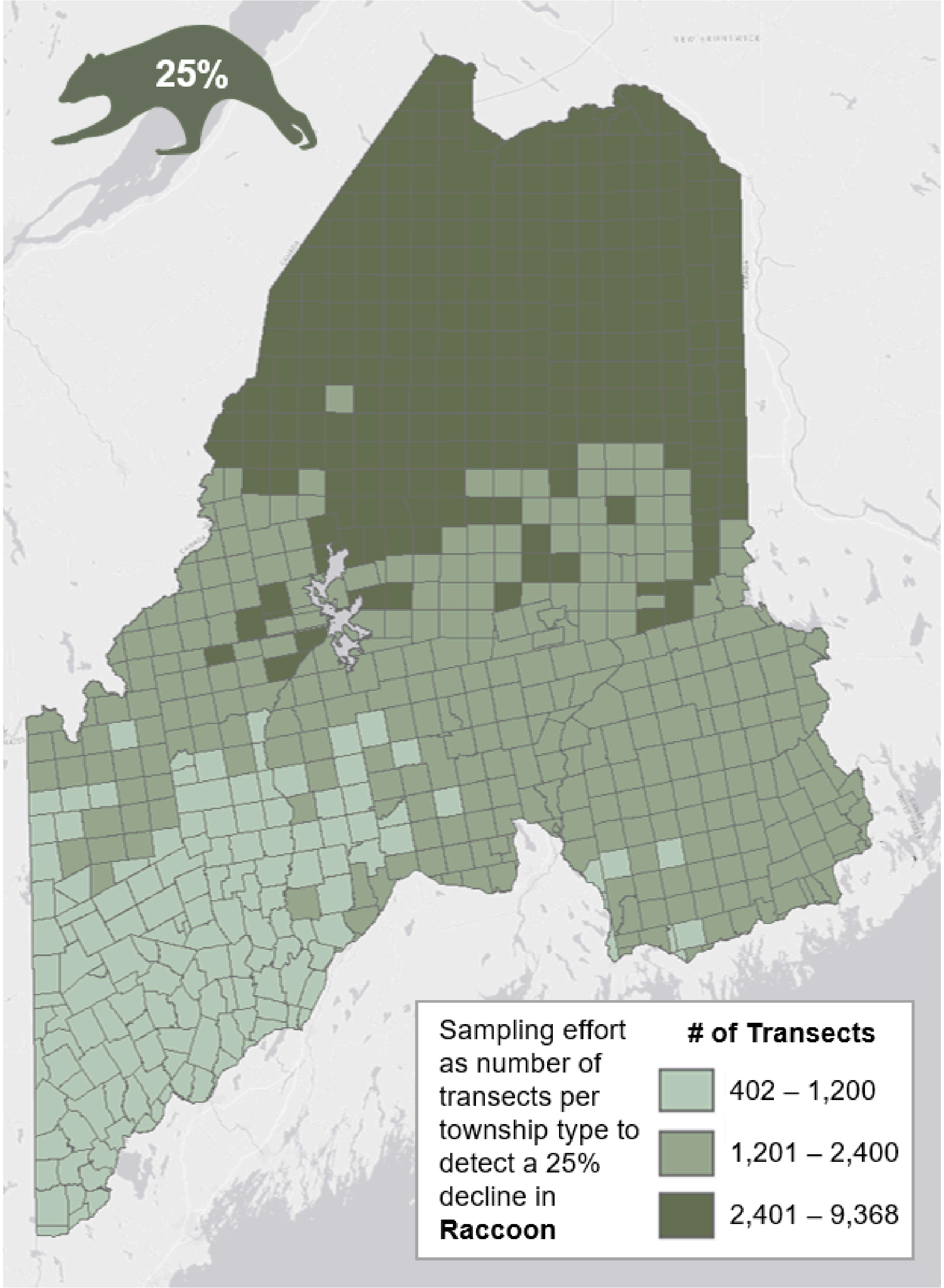

Fig. S3m) Survey effort for raccoon in terms of the number of transects to be deployed across four categories of townships in Maine, USA. Sampling effort refers to the total number of transects to be deployed across all townships of the same category, not per township. As an example, to detect a 25% decline of raccoon across all areas colored in lightest sage green, 1,200 transects are required.

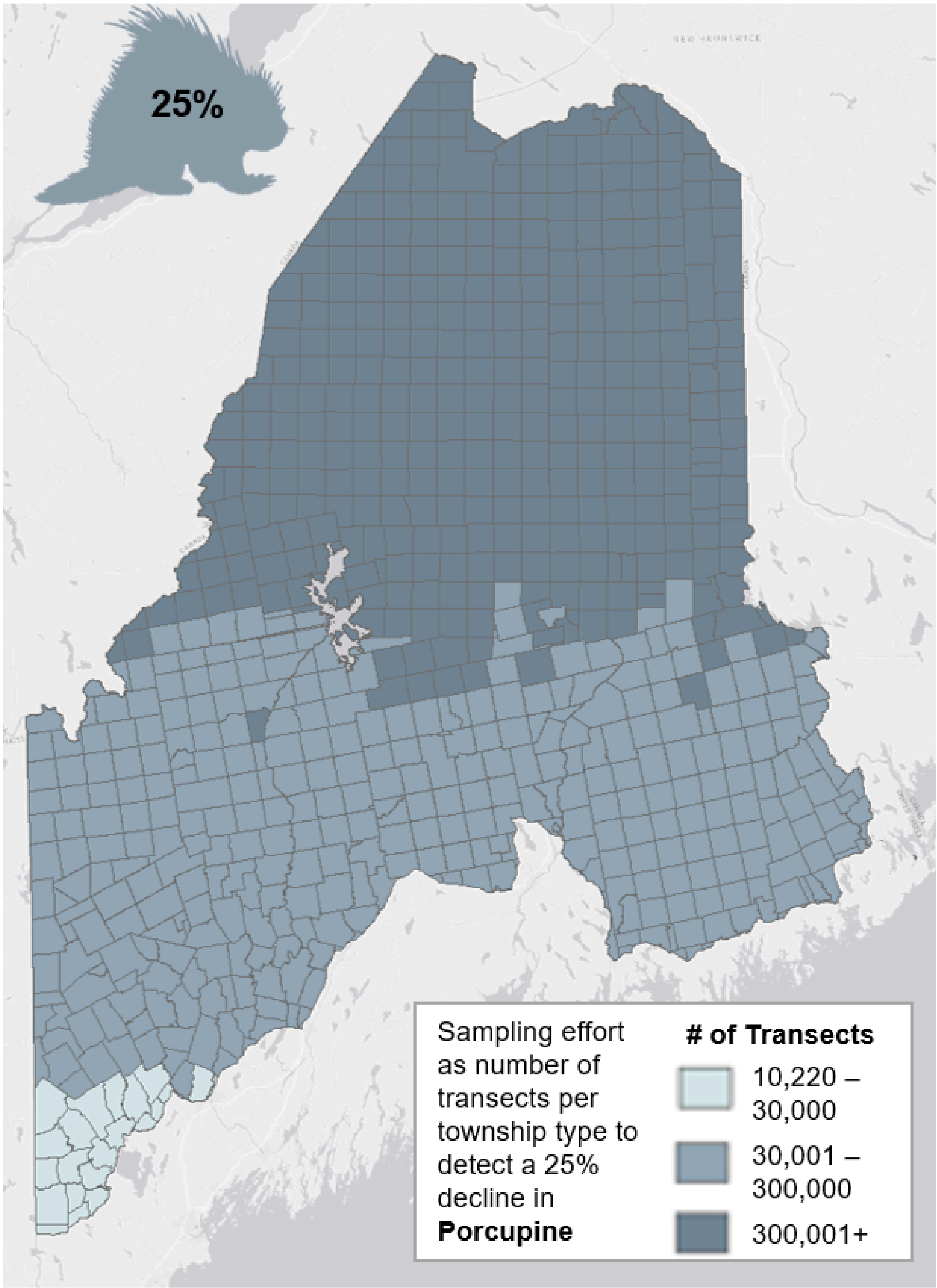

Fig. S3n) Survey effort for North American porcupine in terms of the number of transects to be deployed across four categories of townships in Maine, USA. Sampling effort refers to the total number of transects to be deployed across all townships of the same category, not per township. As an example, to detect a 25% decline of porcupine across all areas colored in lightest gray, 30,000 transects are required.

Supplemental Information: Appendix 1

Case Study: Assessing the effect of an American marten
monitoring protocol as an umbrella for coyote

Objective: To assess the umbrella effect that a monitoring protocol designed to detect a 25% decline in American marten (target species) could have for a second-priority species, coyote, also at 25% decline across American marten habitat in central and northern Maine.

Step 1: *Determine the sampling effort required to monitor for a 25% management objective in the target species (American marten)*

To allocate resources according to our objective, we first need to identify which areas in Maine would require extreme effort, high effort, medium effort, or low effort in order to detect 25% declines *in those areas*. Here we describe methods used to achieve this first step.

In order to determine where and how intensively to monitor for species in Maine, we have discretized the continuous habitat using the *township* minor civil divisions already established. For each township in Maine, using occupancy models derived from four years of camera trapping data, we estimated a value for *detection* probability and for average *occupancy* probability (see methods) using the township-level estimates of the following parameters: the average proportion of the forest comprised of *hardwood* trees, the average *disturbance* score (incorporating both how recent and how intense timber harvest activities have been) and the *latitude* at the midpoint. We then used the formulas described in Guillera-Arroita and Lahoz-Monfort (2012) to calculate a single value termed the *survey effort*. As occupancy and detection are calculated at the township-level, survey effort is also calculated for each township. However, this *effort* is, concretely, the number of transects that must be set in all townships with those same values to detect the desired level of population decline. Since multiple townships will have close values (due to close values of hardwood, disturbance, and/or latitude), townships that are similar in survey effort can be grouped together into one *category* and given the same value for effort: to be conservative, we elected to assign the maximum effort needed for any township in the group to all townships in the resulting category. **We emphasize that this effort refers to the maximum number of transects required across all townships of the same category, not per township.**

How townships are grouped into categories is a critical decision point for several reasons: 1) Accurate biological inferences must come from data collected at the correct scale, which will vary by species. Although the input data used to understand the features of occupancy and detection going into the protocol are collected at the

township scale, this is too fine-grain to allow state-wide interpretation. Expert knowledge and critical thinking are then essential to determine the scale that monitoring is both feasible and biologically relevant for the management objectives. 2) The number of categories ultimately determines the total effort required – a trade-off between detecting trends tied to specific combinations of habitat features (more categories will allow more detailed inference, but requires more effort) and the most efficient plan to detect overall population trends (fewer categories, less effort).

How much effort you can expend on camera trapping to reach your objectives will determine the precision of monitoring for the target species, as well as the umbrella effect for additional second-priority species. If you can only afford to deploy very few survey sites, the areas of the state with low habitat suitability / low detectability may not be feasible to monitor. Instead, you may need to focus on only townships in a category requiring up to the number of sites you can deploy, and then the trends you detect would reflect populations in these habitat types only.

For marten, we chose to examine four categories, defined along the natural jenks in the spatial dataset, as shown below (and Fig. A1.1):

Low effort: require up to 462 total survey sites
Medium effort: require up to 463-693 sites
High effort: require up to 694-1,155 sites

To effectively monitor all three categories requires a total of 1,115 camera transects, which we felt was unreasonable for a state management agency. Instead, the map below shows a hypothetical distribution of camera transects in the lower two categories – these are townships where the habitat features that were most important for describing marten occupancy patterns and detectability create easy and moderate conditions for monitoring marten. Both occupancy and detection for marten were primarily forest disturbance, followed by forest composition (Fig. A1.1).

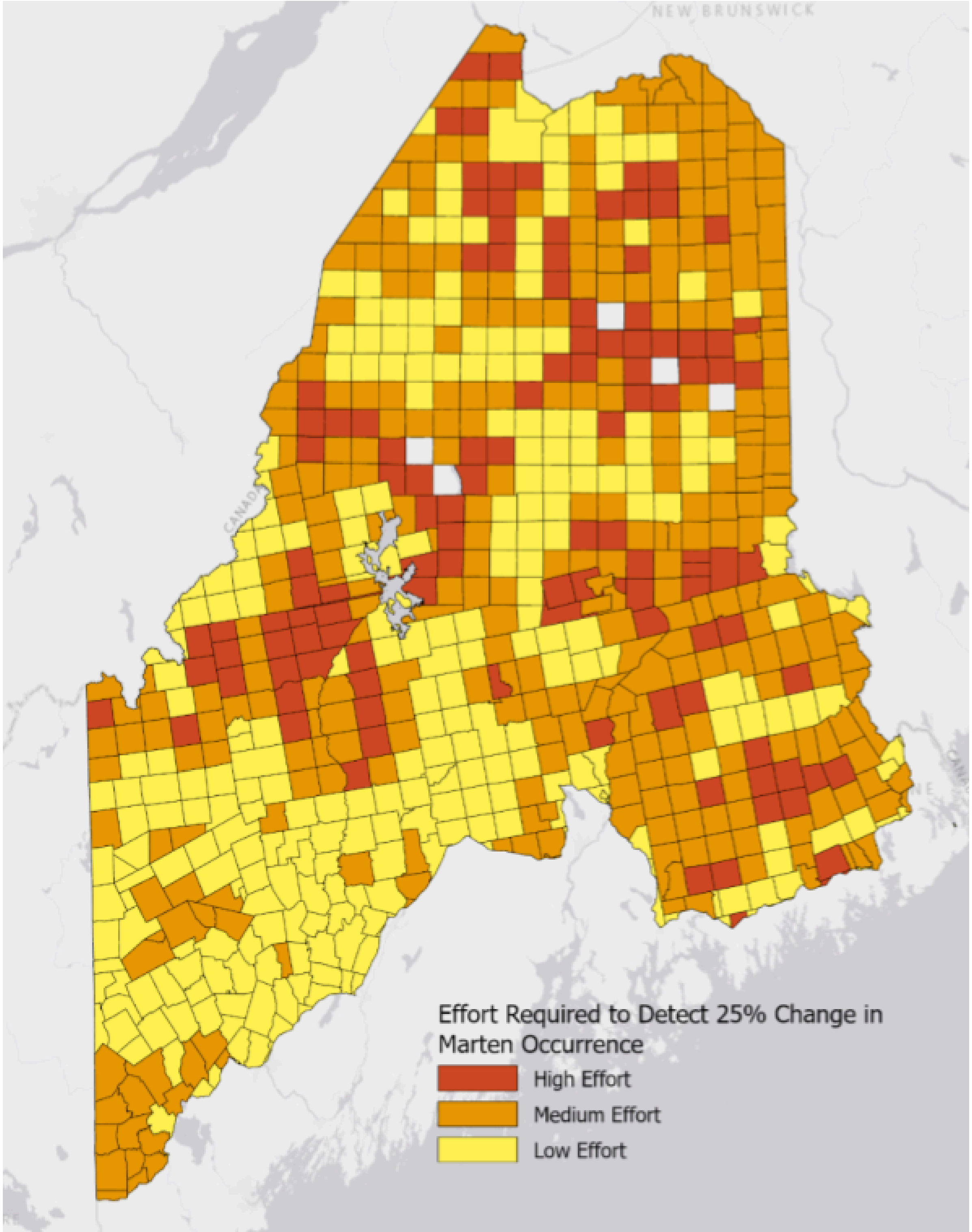

Fig. A1.1. Map of the sampling effort required to detect 25% change in occupancy for American marten across three township categories. Low effort requires up to 462 transects, medium effort requires up to 693, and high effort 1,155.

Step 2*: Decide where to allocate the required sampling effort in order to achieve an optimal umbrella effect for the second-priority species (coyote).*

If only American marten were considered a priority species, the next step would be to establish the correct number of survey locations across any of the townships in each category. However, because coyote are also a species of interest, our next step is to identify which townships, at the survey effort needed for marten, also cover the survey effort needed to detect a 25% decline in coyote.

Coyote occupancy and detection patterns were driven by latitude and were most easily detected in the south of the state (see Fig. S2c and S3d). The three maps below (Fig. A1.2 a-c) show the townships with survey effort for detecting 25% marten declines (solid color) at 462, 693 and 1,155 camera transects that would *also* provide reliable data for coyote (hashed lines). Note that townships in the far north are never covered under the umbrella of the marten monitoring protocol – this is because coyote effort in this areas is higher than the maximum effort for marten.

To monitor all three categories would require 2,311 survey sites, which is unreasonable to ask of a research team or state agency. To monitoring only the regions with low and moderate effort needed to detect 25% change, we would require 1,156 total transects, with 463 distributed in low effort townships and 693 in high effort townships (Fig A1.3).

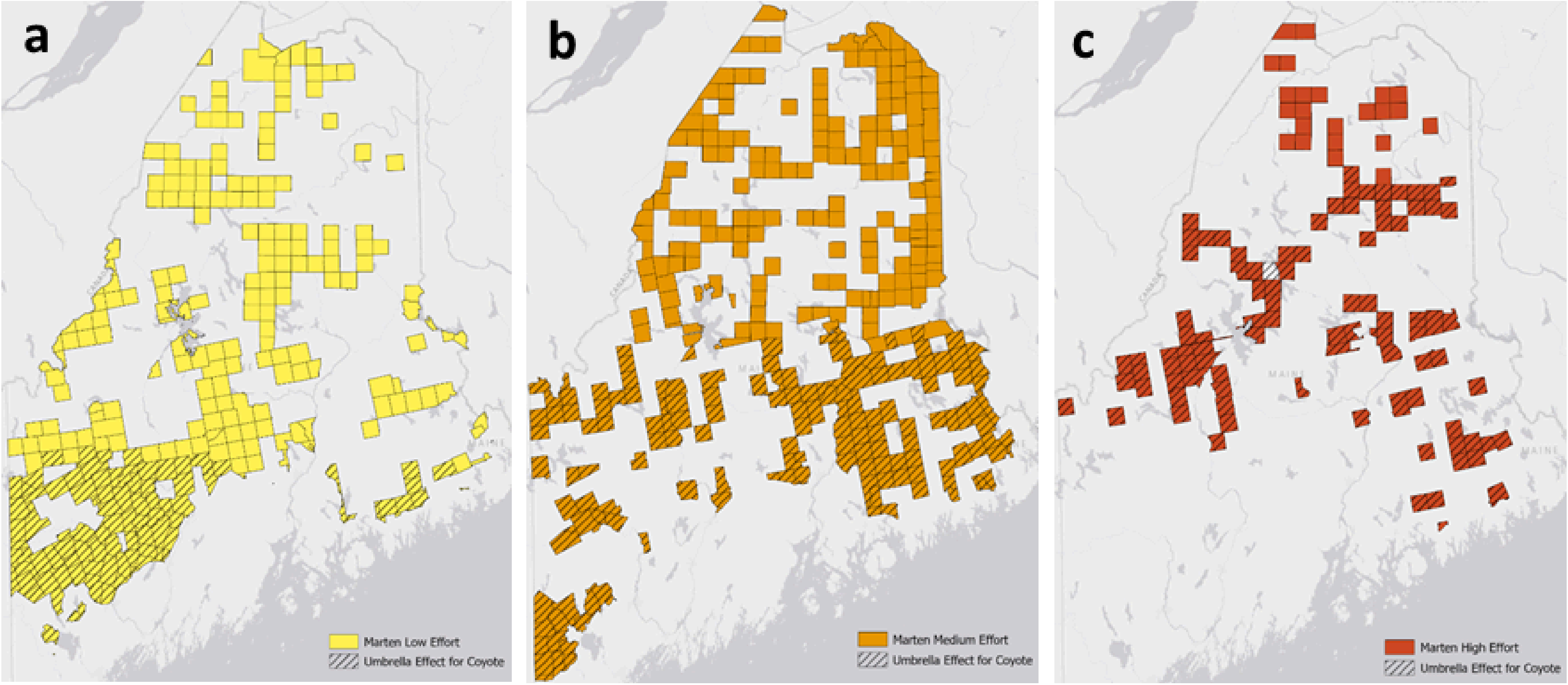

Fig. A1.2. (a) Low effort townships (463 survey stations) that would detect 25% declines for marten (solid yellow) and 25% declines for coyote (hashed). (b) Medium effort townships (693 survey stations) that would detect 25% declines for marten (solid orange) and 25% declines for coyote (hashed). (c) High effort townships (1,155 survey stations) that would detect 25% declines for marten (solid red) and 25% declines for coyote (hashed).

Compiling all of this information together, we can create the map below (Fig. A1.3) and plan the locations of monitoring areas to fall in regions where *both* marten and coyote population trends can be detected.

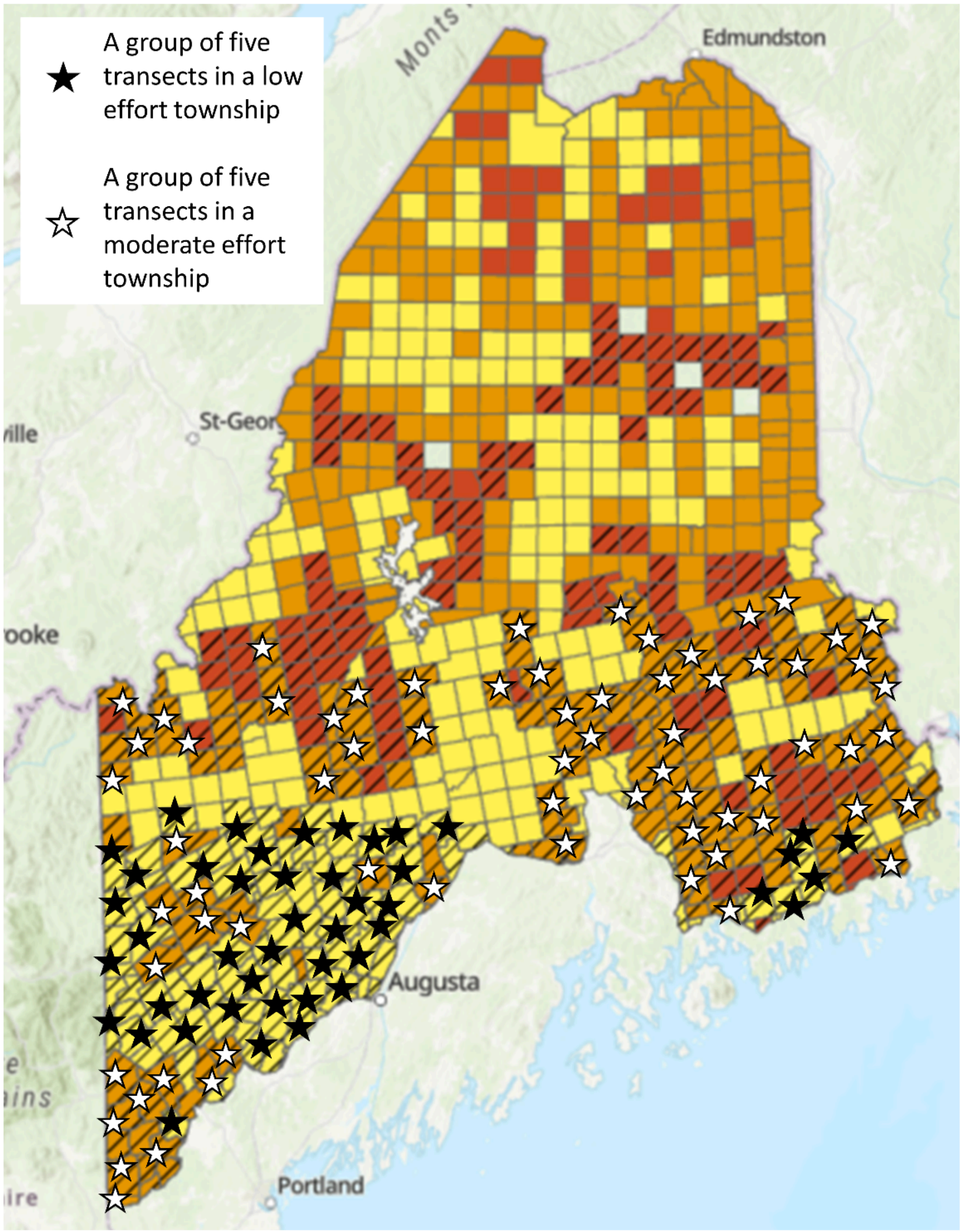

Fig. A1.3. Map of proposed sites for monitoring both marten and coyote to detect a 25% change in regions where low or moderate effort is required for marten.

In Fig A1.3, each star indicates a cluster of five camera transects. Note that to meet model assumptions, transect must be placed at minimum 6 km apart, which is feasible as typically "township" in Maine is 100 km^2 in area, assuming that all land is accessible. Black stars are on townships eligible for monitoring at the lower effort threshold for marten (463 total transects) and where coyote monitoring needs are the same or lower. White stars are on townships where moderate effort is required for marten (693 total transects) and the same or lower required for coyote.

Step 3: *Interpreting results and translating into management objectives*

With 1,156 transects as a goal for total effort, we demonstrate above that we can distribute these across 1) low and medium effort areas for American marten that simultaneous 2) provide an umbrella effect for coyote. Once the final survey locations are established, we would then be able to analyze all of the opportunistic data collected on all other species in light of each one's unique monitoring requirements. For example, black bear are relatively easy to obtain high precision change detection, so almost any combination of survey points will provide reliable information on their population trends. Other species such as lynx, which was not specifically targeted in this example, may still provide some indication of trends at a coarser scale, and the degree of uncertainty can be better quantified by using our multi-species umbrella monitoring framework.

Finally, it is critical to interpret the results of these monitoring plans appropriately. In this example, our top priority was to detect a 25% decline in American marten, across all of their habitat types excepting only the <1% of the very poorest quality (and hardest to monitor). With 1,156 stations, we are able to meet this primary objective. As a secondary objective, we wanted to also track 25% declines in coyote populations. With the limitations of 1,156 stations, the farthest north of the state was not possible to survey. Thus, while we were able to meet the monitoring requirements for the southern two-thirds of the total potential area, it is important to acknowledge this limitation when interpreting results and making management decisions. Because the biology of the species is at play, we cannot extrapolate trends observed in the southern portion to the entire state.

Supplemental Information: Appendix 2

Examples of camera trap images for the 14 mammalian species included in our analyses, recorded on Bushnell Trophy Cam E2and E3 models, deployed across 197 sites in Maine, USA, in summer and winter from June 2017 to October 2020.

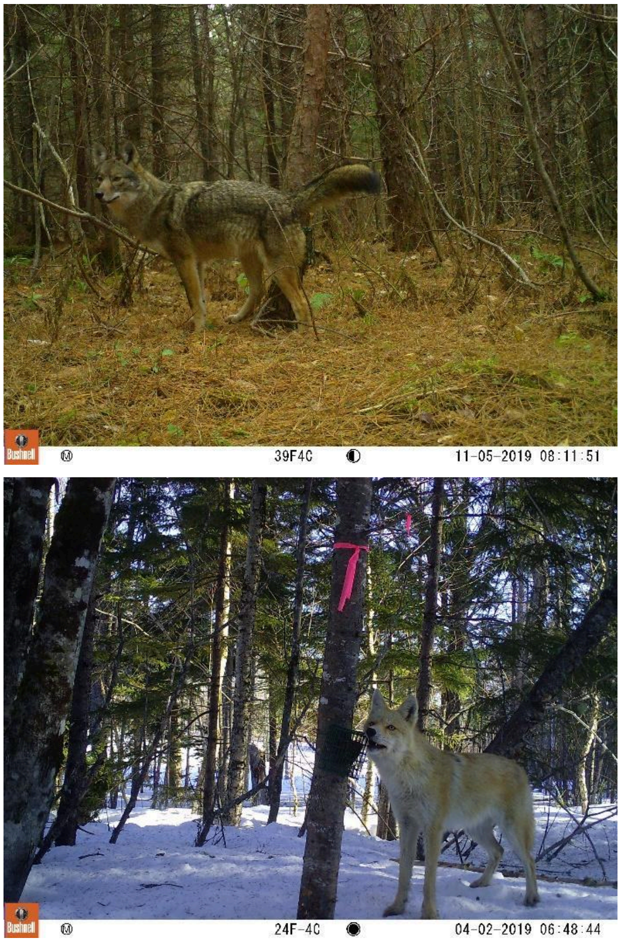

Fig. A2a. Coyote (*Canis latrans*)

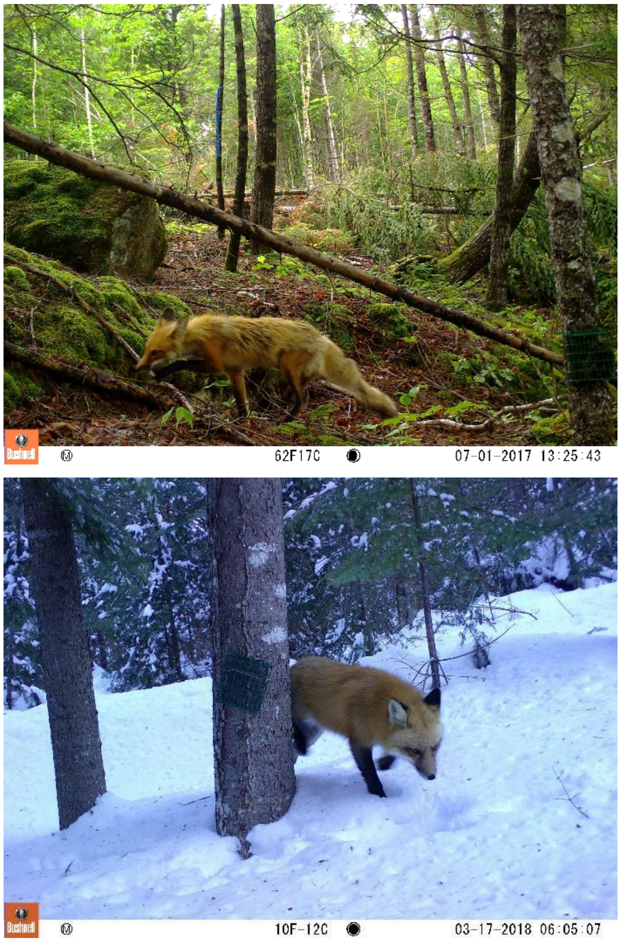

Fig. A2b. Red fox (*Vulpes vulpes*)

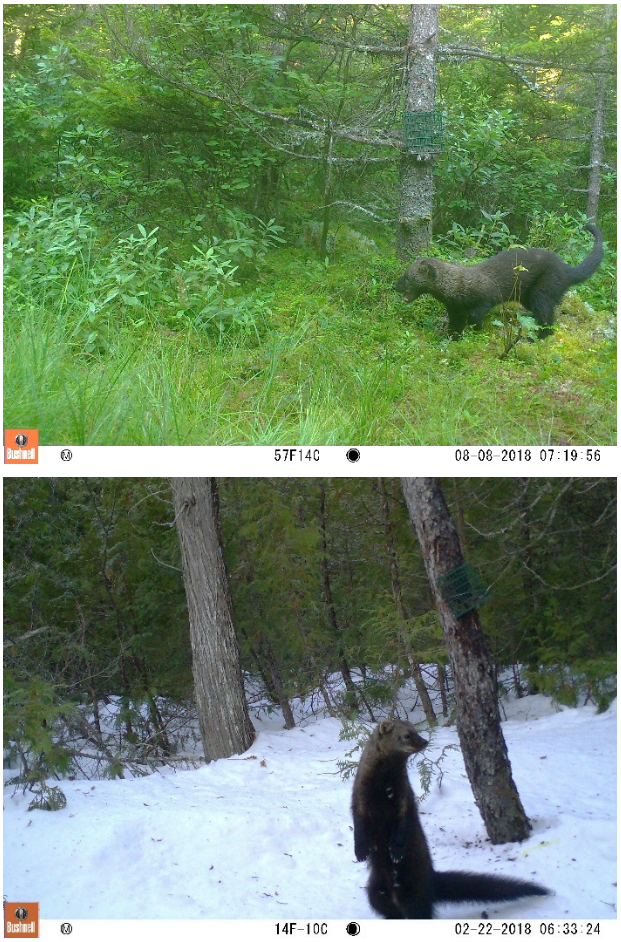

Fig. A2c. Fisher (*Pekania pennanti*)

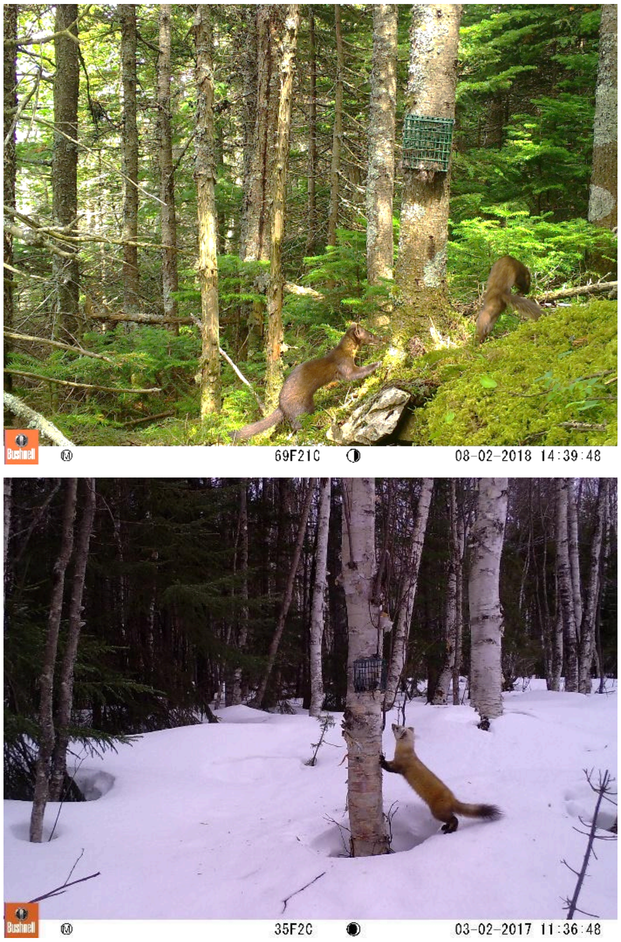

Fig. A2d. American marten (*Martes americana*)

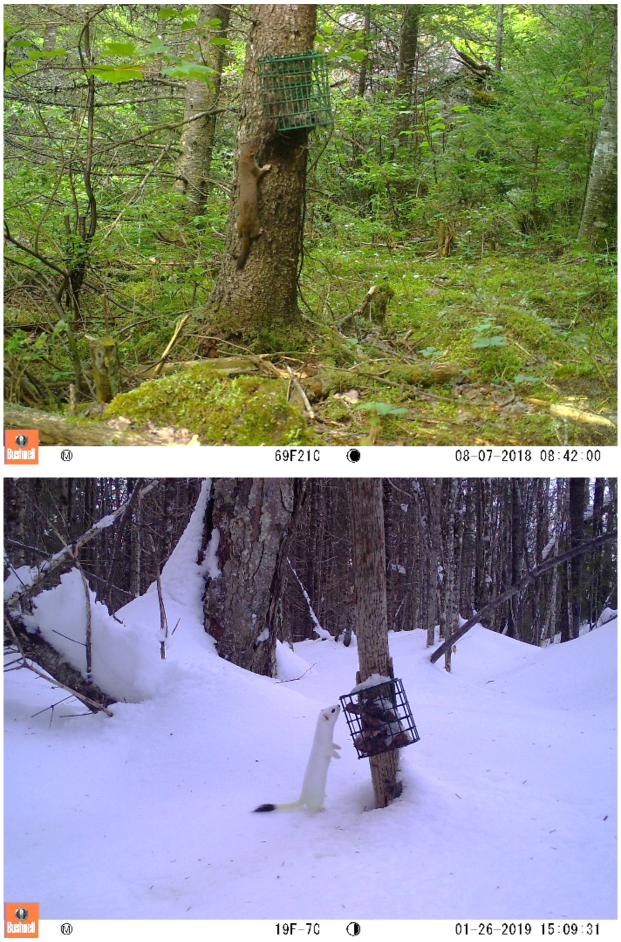

Fig. A2e. Short-tailed weasel (*Mustela erminea*)

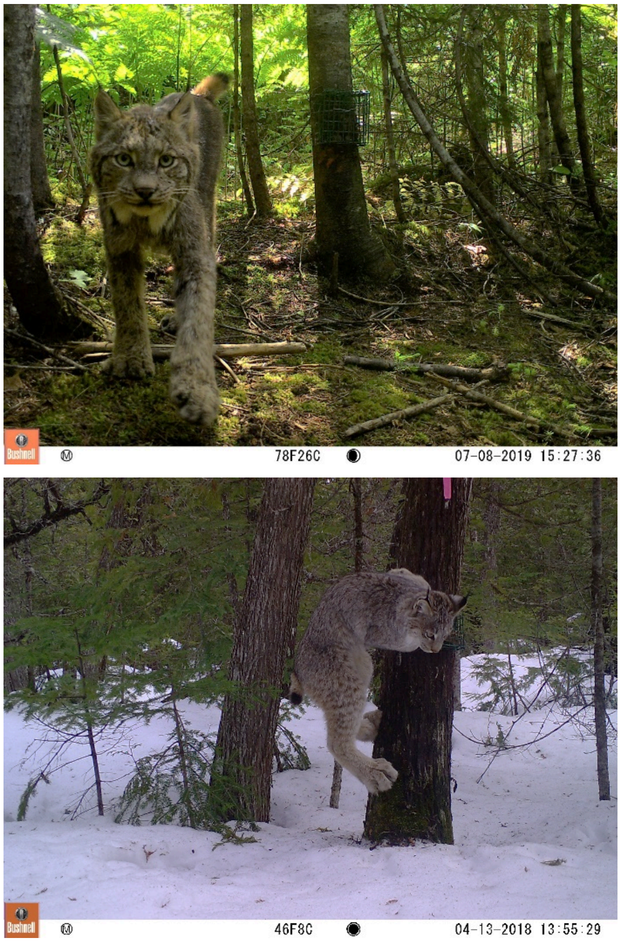

Fig. A2f. Lynx (*Lynx canadensis*)

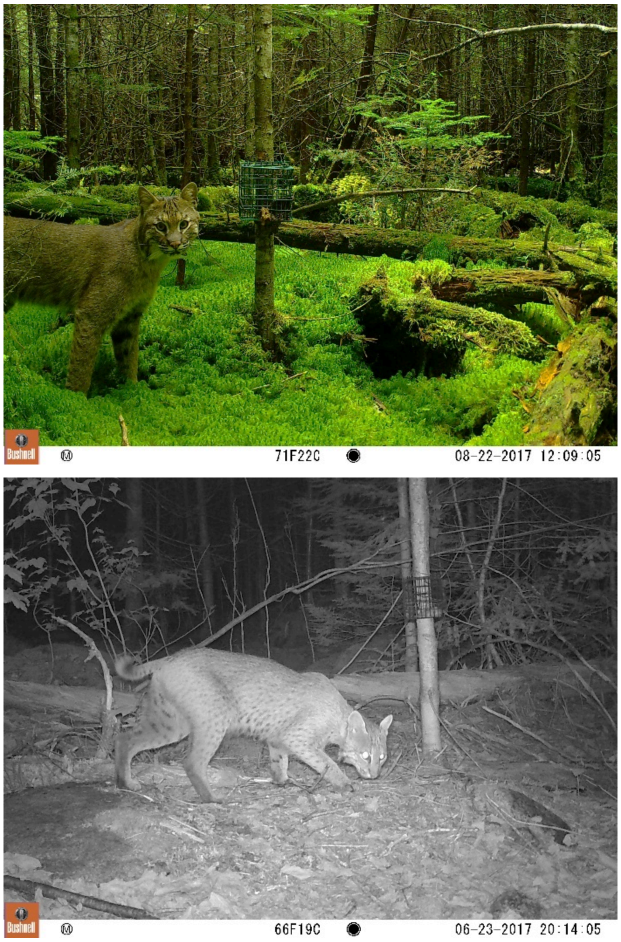

Fig. A2g. Bobcat (*Lynx rufus*)

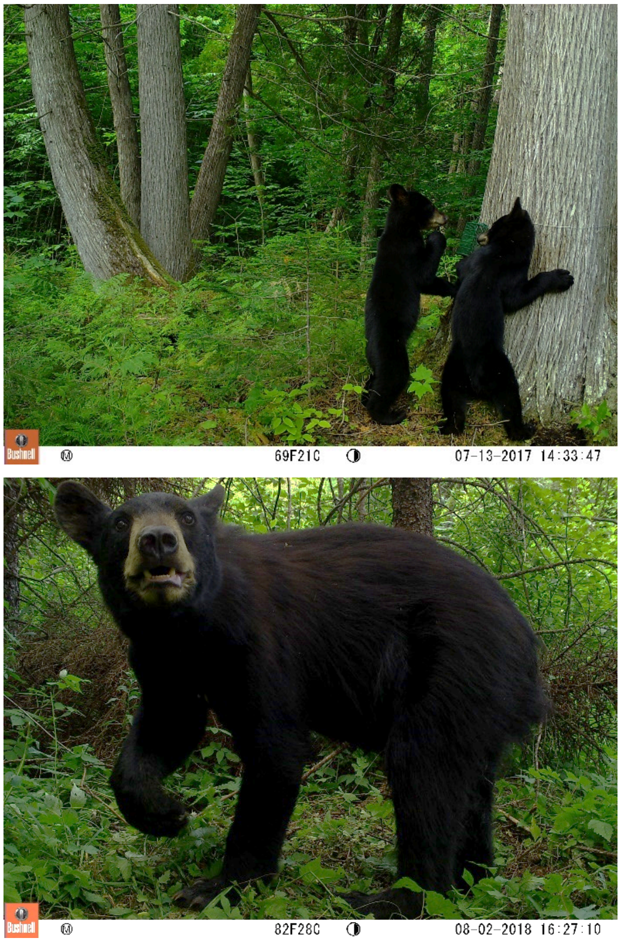

Fig. A2h. American black bear (*Ursus americanus*)

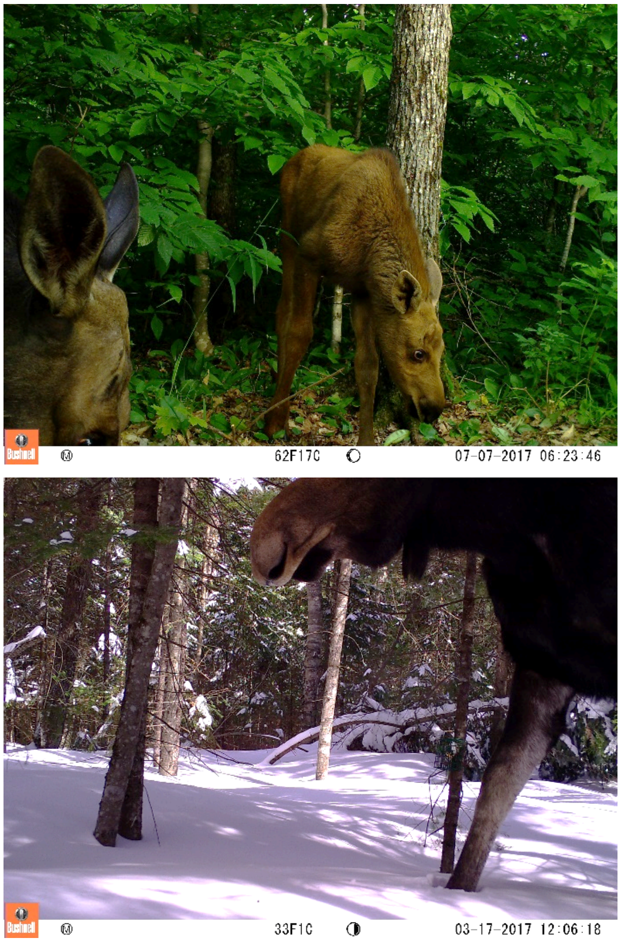

Fig. A2i. Moose (*Alces alces*)

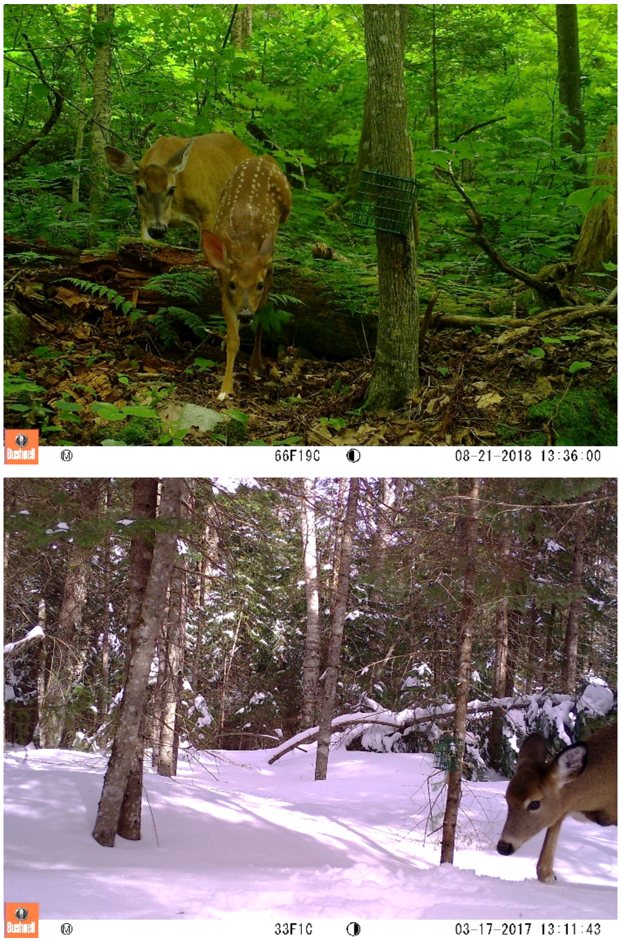

Fig. A2j. White tailed deer (*Odocoileus virginianus*)

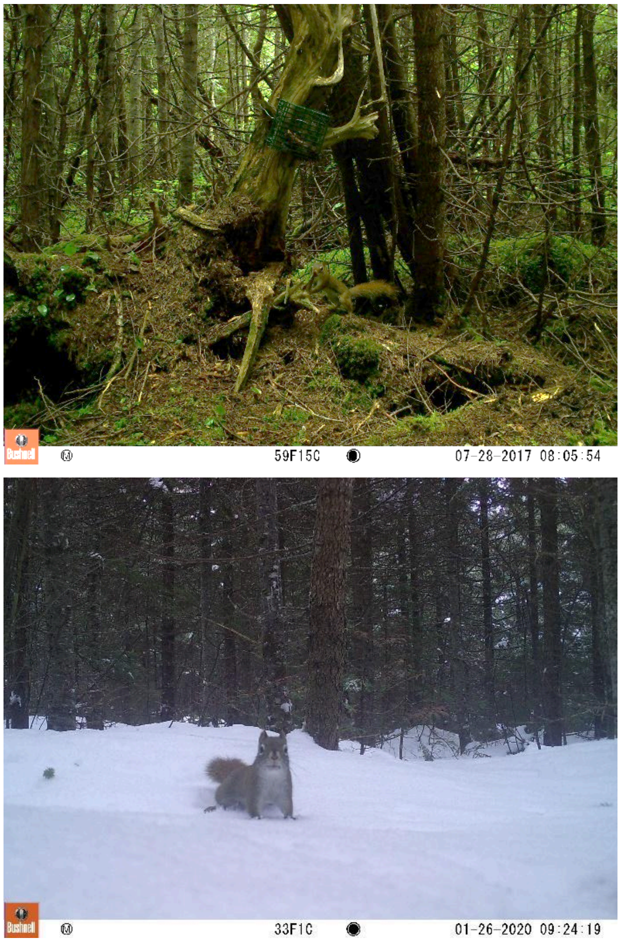

Fig. A2k. American red squirrel (*Tamiasciurus hudsonicus*)

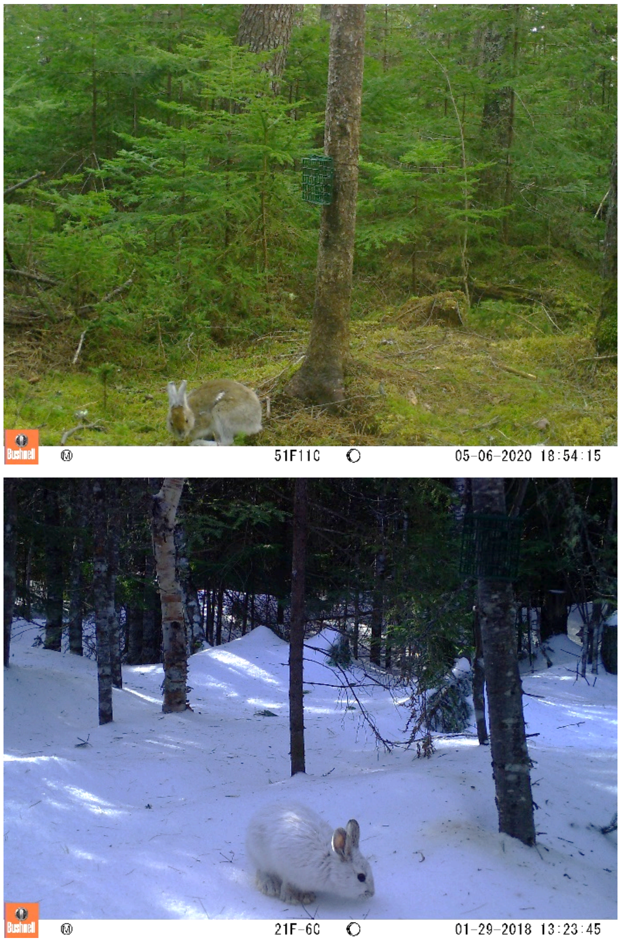

Fig. A2l. Snowshoe hare (*Lepus canadensis*)

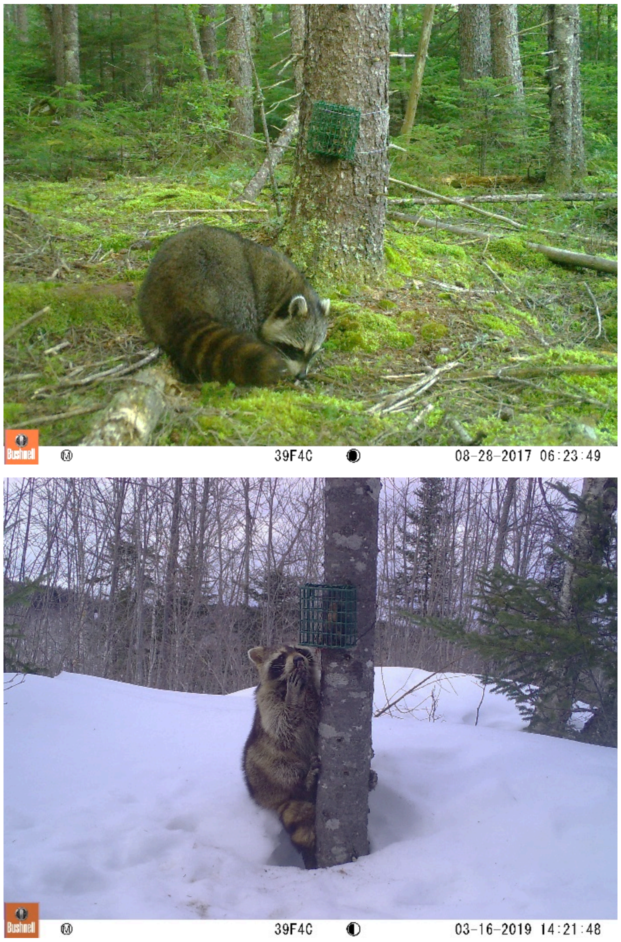

Fig. A2m. Raccoon (*Procyon lotor*)

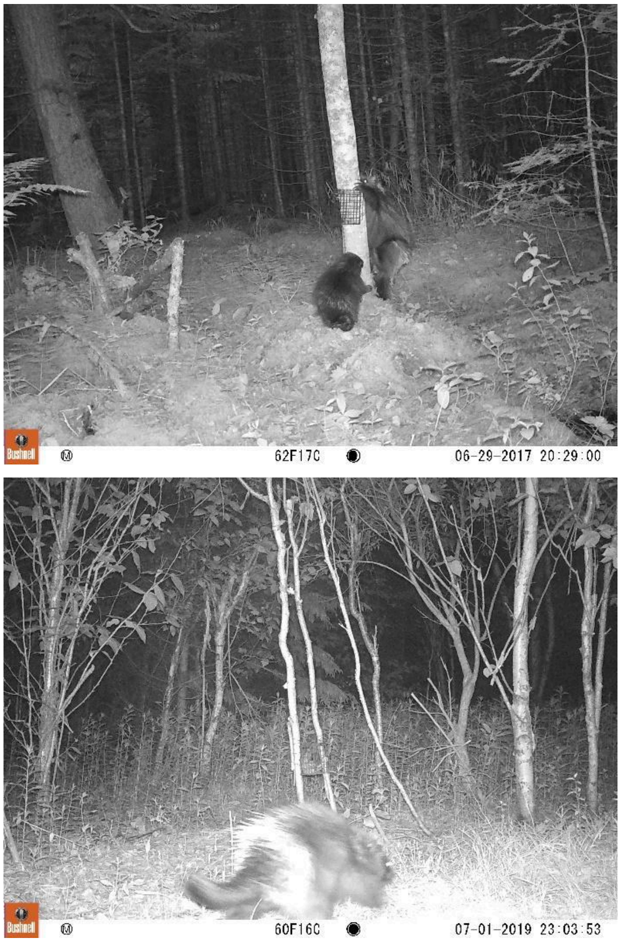

Fig. A2n. North American porcupine (*Erethizon dorsatum*)
